# Phytochemical Profile and Cosmeceutical Potential of *Lotus germanicus*: Antioxidant, Anti-Tyrosinase, Anti-Elastase and Antibacterial Activities of Parent Plant and In Vitro Cultures

**DOI:** 10.3390/molecules31213940

**Published:** 2026-11-01

**Authors:** Marta Klimek-Szczykutowicz, Katarzyna Kulik-Siarek, Jakub Wawrzycki, Paulina Żarnowiec, Karolina Wiśniewska, Renata Piwowarczyk, Agnieszka Szopa

**Affiliations:** 1 Department of Pharmaceutical Sciences, Collegium Medicum, Jan Kochanowski University of Kielce, IX Wieków Kielc 19a, 25-516 Kielce, Poland; katarzyna.kulik-siarek@ujk.edu.pl (K.K.-S.); jakub.wawrzycki@ujk.edu.pl (J.W.); 2 Department of Microbiology, Institute of Biology, Jan Kochanowski University of Kielce, Uniwersytecka 7, 25-406 Kielce, Poland; paulina.zarnowiec@ujk.edu.pl; 3 Center for Research and Conservation of Biodiversity, Department of Environmental Biology, Institute of Biology, Jan Kochanowski University of Kielce, Uniwersytecka 7, 25-406 Kielce, Poland; karolina.ruraz@ujk.edu.pl (K.W.); renata.piwowarczyk@ujk.edu.pl (R.P.); 4 Department of Medicinal Plant and Mushroom Biotechnology, Faculty of Pharmacy, Jagiellonian University Medical College, Medyczna 9, 30-688 Kraków, Poland

**Keywords:** *Lotus germanicus*, *Dorycnium germanicum*, plant in vitro cultures, polyphenolic profile, antioxidant potential, enzyme inhibition, antibacterial activity

## Abstract

*Lotus germanicus* (syn. *Dorycnium germanicum*; Fabaceae) is a rare and regionally threatened European species that remains poorly investigated phytochemically and biologically. This study provides a comprehensive characterization of its phytochemical profile and biological activity and, to our knowledge, the first evaluation of in vitro cultures within the genus *Lotus* (formerly classified under *Dorycnium*). Extracts from leaf, herb, and microshoot cultures were analyzed by HPLC-DAD and biological assays. Phenolic acids, flavonoids, sterol-related compounds, and triterpenoids were identified. Isoquercitrin was the predominant flavonoid (5860.00 mg/100 g DW), while hydrocaffeic acid was the major phenolic acid (575.53 mg/100 g DW). In microshoot cultures, β-sitosterol and ursolic acid reached 152.94 and 146.30 mg/100 g DW, respectively. Parent-plant extracts contained higher total phenolic and flavonoid levels and generally showed stronger antioxidant, anti-tyrosinase, and anti-elastase activities. All extracts exhibited antioxidant activity and selective antibacterial activity, mainly against Gram-positive bacteria, with MIC values ranging from 5 to 20 mg/mL for susceptible strains. Despite lower metabolite levels, microshoot cultures retained measurable biological activity. These findings suggest that *L. germanicus* may represent a potential source of bioactive compounds for further investigation in cosmetic and dermatological applications and highlight the potential of *Lotus* sect. *Dorycnium* in vitro cultures as an alternative source of plant-derived metabolites. However, further studies using skin-relevant biological models and formulated cosmetic products are required to confirm the efficacy and safety of these extracts before their practical application.

## 1. Introduction

The genus *Lotus* L., belonging to the tribe Loteae within the Fabaceae family, is one of the largest and most taxonomically complex genera of this tribe. Species traditionally classified within *Dorycnium* Mill. were historically treated as a separate genus, mainly distributed in the Mediterranean region and Macaronesia. However, molecular phylogenetic and morphological studies have shown that *Dorycnium* cannot be clearly separated from *Lotus*, and contemporary taxonomic treatments generally include former *Dorycnium* species within an expanded genus *Lotus*. Chemotaxonomic similarities, particularly the occurrence of myricetin derivatives, 5-deoxyflavonols, and 8-*O*-substituted flavonols of the sexangularetin type, further support the close relationship between these groups [1,2,3]. One representative of this group is *Lotus germanicus* (Gremli) Peruzzi, with *Dorycnium germanicum* (Gremli) Rikli regarded as a taxonomic synonym. The species is a perennial herbaceous to suffrutescent plant or small shrub occurring mainly in Central and Southeastern Europe, including Austria, Bulgaria, Germany, Greece, Hungary, Italy, Poland, Romania, Switzerland, and parts of the Balkan Peninsula. It develops stems approximately 10–50 cm long, with predominantly appressed pubescence, oblong to obovate leaflets, and long-pedunculate inflorescences comprising several flowers [1,2]. *L. germanicus* is a relatively rare species in Central Europe and has been included in several national and regional Red Lists. It is classified as Vulnerable in Switzerland [4] and Germany [5], while in the Czech Republic it is listed in category C3, corresponding to vulnerable taxa [6]. In Austria, the species is also considered of conservation concern and has been classified as Near Threatened in regional assessments, including the Red List of Burgenland. In Poland, *L. germanicus* is a rare and threatened species included in the Polish Red Data Book of Plants and classified as endangered [7]. The conservation status of *L. germanicus* highlights the importance of limiting the collection of plant material from natural populations. In this context, plant in vitro cultures may provide a sustainable alternative source of biomass and secondary metabolites, allowing for phytochemical and biological investigations while reducing pressure on naturally occurring populations.

Previous phytochemical investigations of other *Dorycnium* species indicate that representatives of this group may constitute valuable sources of bioactive secondary metabolites, particularly flavonoids. Kaempferol- and quercetin-derived compounds have been identified in *D. hirsutum* [8], while *D. herbaceum* has been reported to contain the isoflavone genistein [9]. The phytochemical profile of *D. rectum* includes epigallocatechin, gallocatechin, catechin, and several kaempferol derivatives [10,11]. In *D. graecum*, myricetin, myricitrin, kaempferol, quercetin, β-sitosterol, and ursolic acid have been identified [12,13]. These findings demonstrate considerable chemical diversity within the genus and indicate that species of *Lotus* sect. *Dorycnium* may represent promising sources of biologically active secondary metabolites. The phytochemical diversity of the genus is accompanied by a broad spectrum of reported biological activities. Extracts and isolated compounds obtained from different *Dorycnium* species have demonstrated antioxidant, antibacterial, antifungal, antiparasitic, anti-inflammatory, anthelmintic, and cytotoxic effects [14,15,16,17,18,19]. Several species have also been used in traditional medicine. For example, aerial parts of *D. rectum* have been used for the treatment of burns and wounds [20], as well as musculoskeletal, cardiovascular, digestive, and skin disorders [21], whereas *D. pentaphyllum* has been used as an antidiarrheal remedy [22]. *D. pentaphyllum* and *D. graecum* have also been traditionally applied in the treatment of hemorrhoidal disorders [12,23].

Despite the available information on other representatives of section *Dorycnium* in the genus *Lotus, L. germanicus* remains largely unexplored from both phytochemical and biological perspectives. To the best of our knowledge, no comprehensive studies have characterized its secondary metabolite profile or biological activity, and no previous reports have investigated in vitro cultures of this species as a potential source of bioactive compounds. Therefore, the aim of the present study was to characterize the phytochemical composition and biological activity of extracts obtained from the leaves and herb of the parent plant and from in vitro microshoot cultures of *L. germanicus*. Particular emphasis was placed on the evaluation of antioxidant activity and inhibition of elastase and tyrosinase, enzymes relevant to skin aging and pigmentation. In addition, the influence of microshoot culture duration on secondary metabolite accumulation and biological activity was investigated to assess the potential of in vitro cultures as an alternative and sustainable source of bioactive compounds.

## 2. Results

### 2.1. Microshoot Appearance

**Figure 1:**
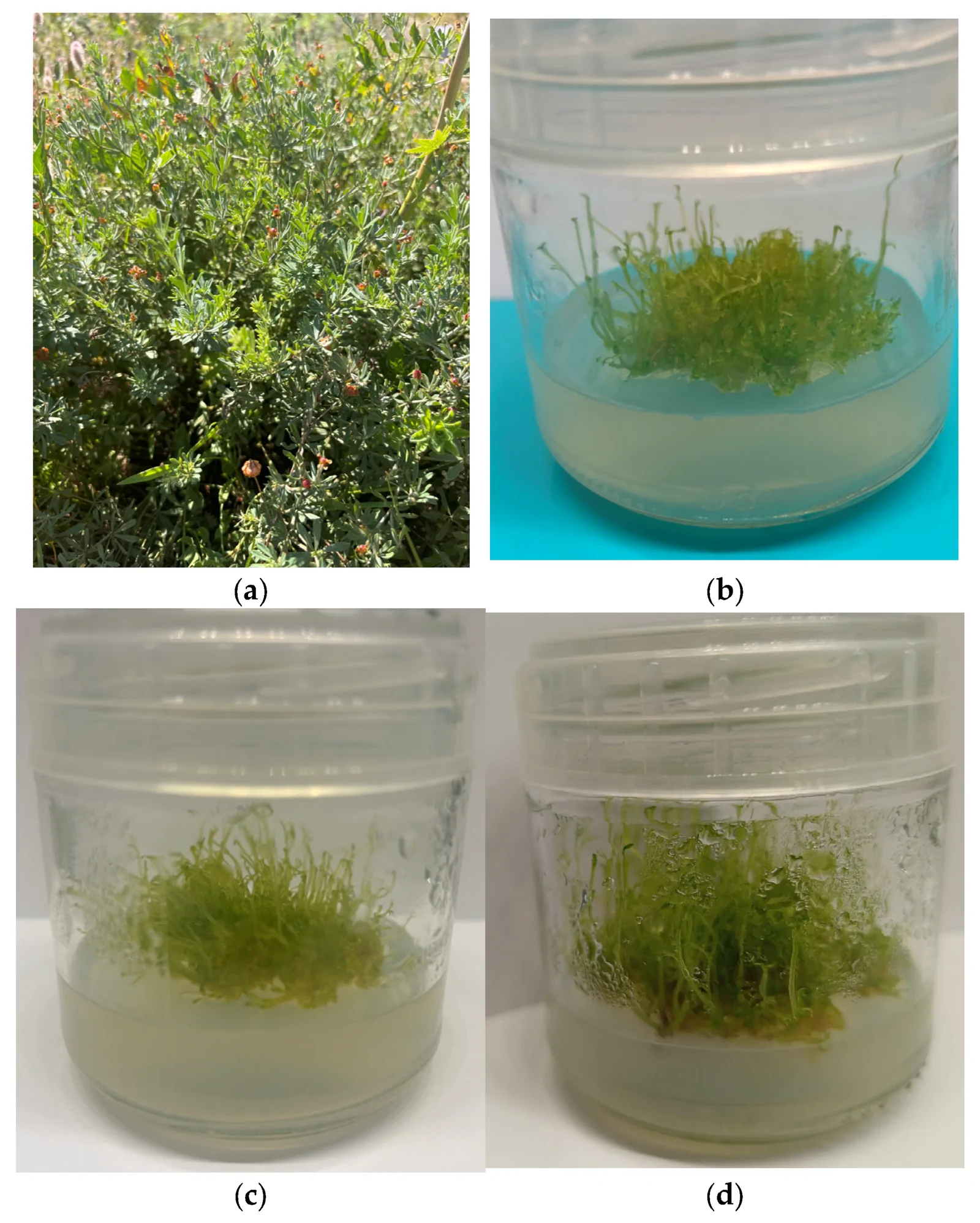
*L. germanicus*: (**a**) general habit; microshoot cultures grown on MS medium supplemented with 1 mg/L BA, 0.5 mg/L NAA, and 0.25 mg/L GA_3_ after (**b**) 10, (**c**) 20, and (**d**) 30 days of cultivation.

Microshoot cultures of *L. germanicus* began germinating 14 days after seed sterilization. The medium for initiation and further growth was MS medium containing 1 mg/L BA (6-benzyladenine), 0.5 mg/L NAA (1-naphthaleneacetic acid) and 0.25 mg/L GA_3_ (gibberellic acid). Cultures were harvested after 10, 20, and 30 days of cultivation (Figure 1b–d). At all sampling times, they were characterized by dense, dark-green microshoots and rapid growth. As the cultivation period progressed, the shoots gradually increased in length. However, after 30 days, the shoots began to show noticeable yellowing.

### 2.2. Phytochemical Profile and Total Phenolic and Flavonoid Contents

The total polyphenol content (TPC) in extracts obtained from leaf, herb, and microshoot cultures of *L. germanicus* was measured using the Folin–Ciocalteu method (Table 1). Herb extracts exhibited the highest total polyphenol content (260.98 mg gallic acid equivalent (GAE)/100 g DW (dried weight)), while a slightly lower value was recorded for leaf extracts (228.52 mg GAE/100 g DW). The lowest polyphenol levels were observed in extracts from microshoot cultures, ranging from 27.85 mg GAE/100 g DW after 10 days of cultivation to 32.98 mg GAE/100 g DW after 30 days.

The total flavonoid content (TFC) was also determined (Table 1). The highest flavonoid level was found in leaf extracts (1033.75 mg rutin equivalent (RE)/100 g DW), whereas herb extracts contained 723.61 mg RE/100 g DW. As in the case of total polyphenols, the lowest flavonoid contents were detected in microshoot culture extracts, ranging from 37.22 mg RE/100 g DW after 10 days of cultivation to 60.71 mg RE/100 g DW after 20 days.

The qualitative and quantitative profiles of compounds present in the analyzed extracts were determined using HPLC-DAD (high-performance liquid chromatography with diode-array detection). Three major groups of compounds were identified in leaf, herb, and microshoot culture extracts of *L. germanicus*: phenolic acids, flavonoids, sterol and triterpenoid-related compounds (Table 1). Flavonoids constituted the predominant group of compounds, particularly in leaf and herb extracts (Table 1). Four flavonoids were identified in both leaf and herb extracts, whereas three were detected in microshoot culture extracts. Isoquercitrin was the most abundant flavonoid, reaching 5860.00 mg/100 g DW in herb extracts and a similarly high level of 5109.06 mg/100 g DW in leaf extracts. Trifolin was also present in considerable amounts, with concentrations of 928.50 mg/100 g DW in herb extracts and 733.82 mg/100 g DW in leaf extracts. However, trifolin was not detected in extracts from the microshoot cultures. Among the flavonoids identified in microshoot culture extracts, isoquercitrin showed the highest content, reaching 25.11 mg/100 g DW after 10 days of cultivation.

Five phenolic acids were identified in both leaf and herb extracts, whereas three were detected in microshoot culture extracts (Table 1). Hydrocaffeic acid was the predominant phenolic acid in herb extracts, reaching 575.53 mg/100 g DW, but it was not detected in the microshoot cultures. In microshoot culture extracts, the highest content was recorded for caffeic acid after 30 days of cultivation (51.06 mg/100 g DW). Moreover, isoferulic acid was detected exclusively in the microshoot cultures, with its highest concentration observed after 10 days of cultivation (25.80 mg/100 g DW), whereas it was absent from both herb and leaf extracts.

Two sterol compounds, β-sitosterol and daucosterol, and one pentacyclic triterpenoid, ursolic acid, were identified in the analyzed extracts (Table 1). Daucosterol reached its highest concentration in leaf extracts (230.81 mg/100 g DW). In microshoot culture extracts, comparatively high levels of β-sitosterol and ursolic acid were recorded after 30 days of cultivation, reaching 152.94 and 146.30 mg/100 g DW, respectively.

### 2.3. Assessment of Biological Activity

#### 2.3.1. The Antioxidant Potential Assessed by ABTS and DPPH Scavenging Assays

**Figure 2:**
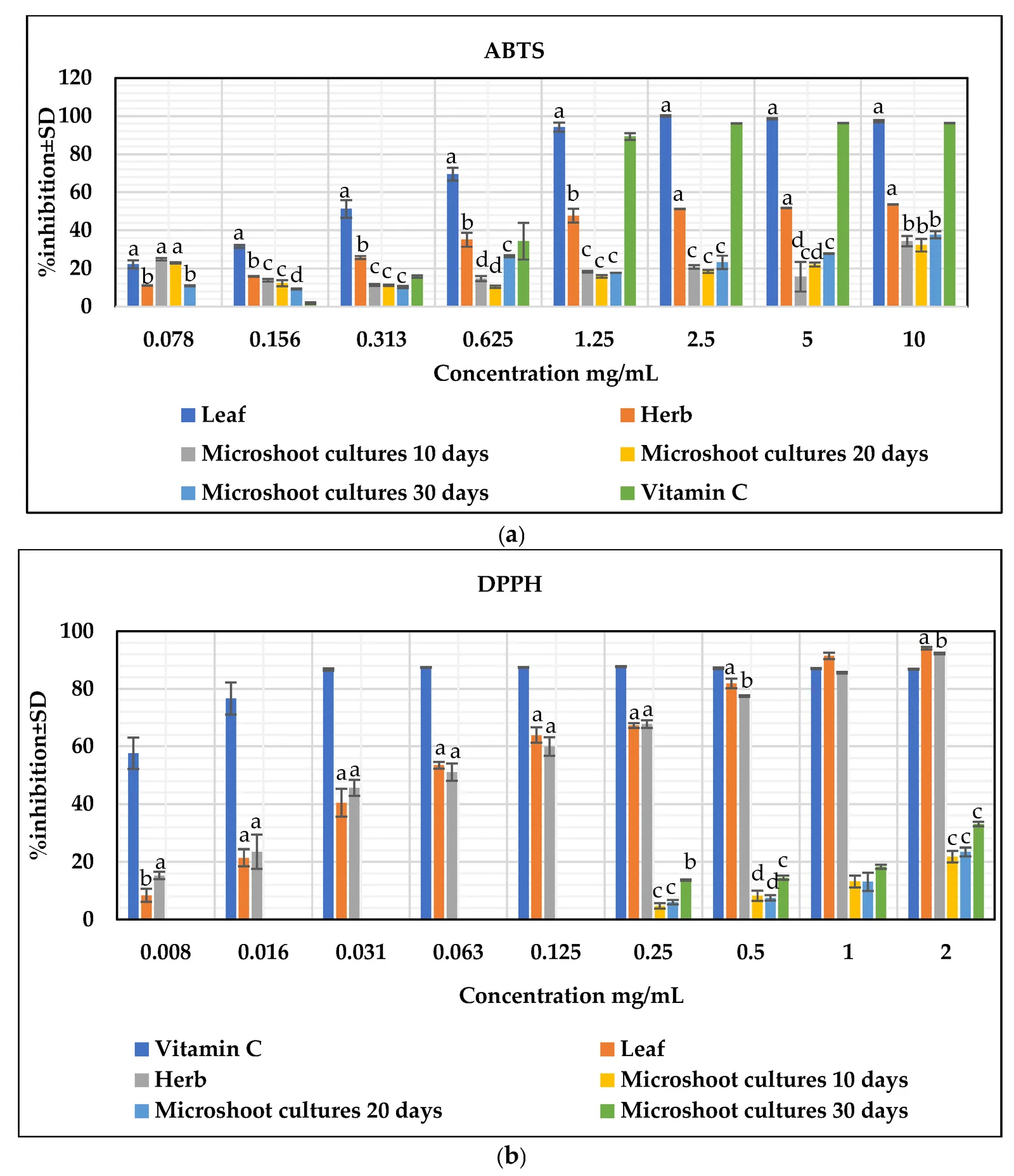
Antioxidant potential (% of oxidation inhibition ± SD), for extracts from leaf, herb and microshoot cultures of *L. germanicus*; (**a**)—ABTS method; (**b**)—DPPH method. Data are presented as mean ± SD of three independent biological replicates (*n* = 3); different lowercase letters indicate statistically significant differences between samples at the same concentration (*p* < 0.05).

The antioxidant potential of *L. germanicus* leaf, herb, and microshoot culture extracts was assessed using the ABTS (2,2′-azinobis(3-ethylbenzothiazoline-6-sulfonic acid) and DPPH (2,2-diphenyl-1-picrylhydrazyl) radical-scavenging assays (Figure 2).

In the ABTS assay (Figure 2a), only the leaf and herb extracts exhibited radical-scavenging activity exceeding 50%. The leaf extract inhibited more than 50% of the ABTS radicals at concentrations of 0.313 mg/mL and higher, whereas the herb extract reached this level of inhibition at concentrations of 2.5 mg/mL and higher (Figure 2a). Accordingly, IC_50_ (half-maximal inhibitory concentration) values were calculated for these extracts. The leaf extract demonstrated the strongest antioxidant activity, with an IC_50_ value of 0.37 ± 0.02 mg/mL, while the IC_50_ value for the herb extract was 0.70 ± 0.05 mg/mL.

The ABTS radical-scavenging activity of the microshoot culture extracts ranged from 10.99% to 37.75%, depending on the extract concentration and cultivation period (Figure 2a). The highest inhibition, 37.75%, was observed for the extract obtained from microshoot cultures after 30 days of cultivation at a concentration of 10 mg/mL. At this concentration, relatively similar results were obtained for extracts collected at different cultivation times, with inhibition ranging from 32.25% for the 20-day microshoot culture extract to 37.75% for the 30-day culture extract. As none of the microshoot culture extracts reached 50% inhibition within the tested concentration range, their IC_50_ values could not be reliably determined.

The DPPH radical-scavenging assay confirmed the antioxidant activity of *L. germanicus* extracts (Figure 2b). Similarly to the ABTS assay, only the leaf and herb extracts achieved more than 50% radical inhibition. Both extracts inhibited more than 50% of the DPPH radicals at concentrations of 0.063 mg/mL and higher. The calculated IC_50_ values were 0.04 ± 0.02 mg/mL for the leaf extract and 0.05 ± 0.02 mg/mL for the herb extract, indicating comparable and pronounced antioxidant activity.

For the microshoot culture extracts, DPPH radical-scavenging activity was evaluated at concentrations ranging from 0.25 to 2 mg/mL, as lower concentrations showed no detectable activity (Figure 2b). Depending on the extract concentration and cultivation period, inhibition ranged from 4.70% to 33.00%. The highest inhibition, 33.00%, was obtained at a concentration of 2 mg/mL for the extract from microshoot cultures collected after 30 days. At this concentration, inhibition values for extracts collected at different cultivation times ranged from 21.76% to 33.00%. As none of the microshoot culture extracts reached 50% inhibition within the tested concentration range, their IC_50_ values could not be reliably determined.

#### 2.3.2. Tyrosinase Inhibitory Potential

**Figure 3:**
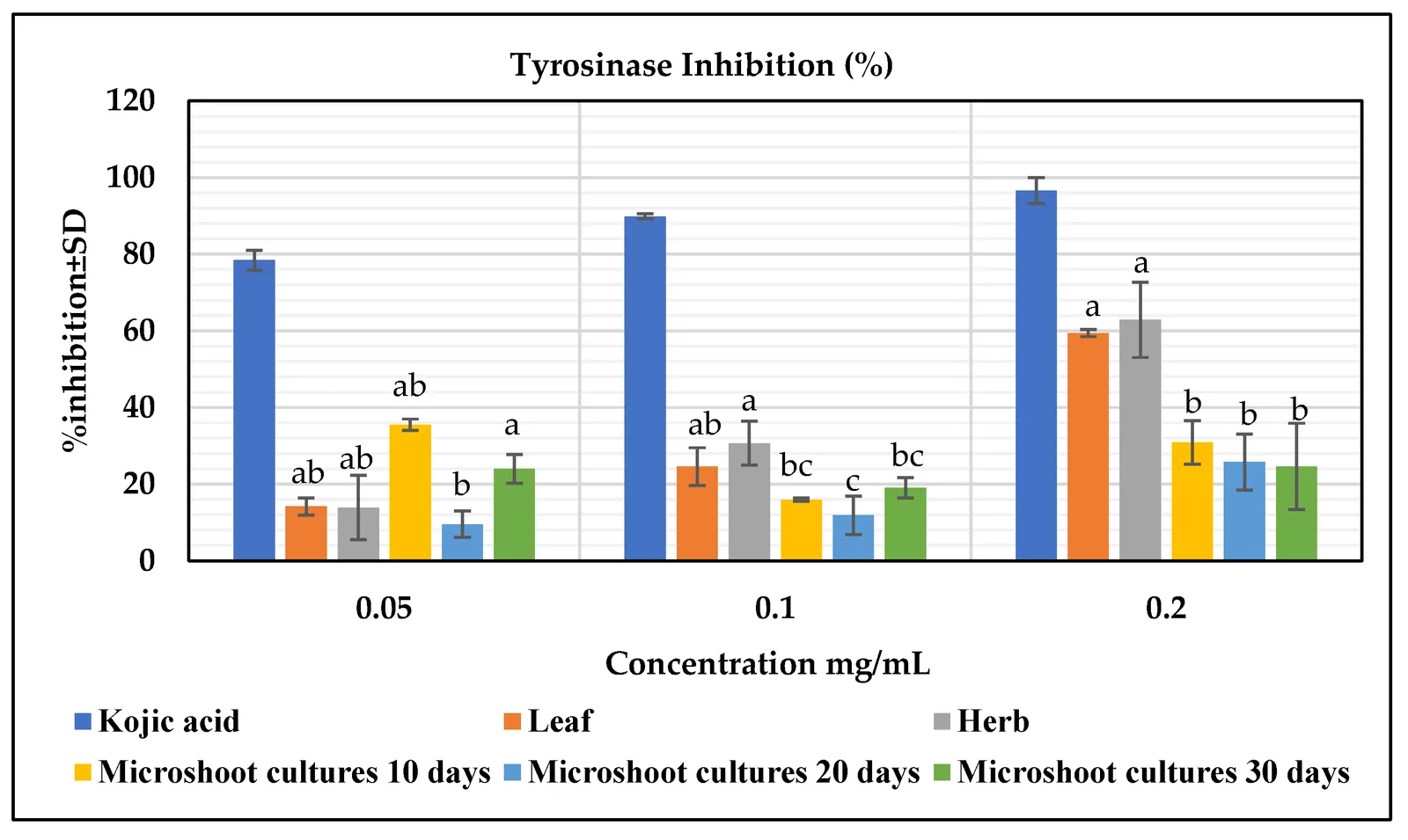
Inhibitory activity of leaf, herb and microshoot cultures of *L. germanicus* extracts on mushroom tyrosinase in comparison with kojic acid. Data are presented as mean ± SD of three independent biological replicates (*n* = 3); different lowercase letters indicate statistically significant differences between samples at the same concentration (*p* < 0.05).

The tyrosinase inhibitory potential of *L. germanicus* extracts was evaluated using commercially available mushroom tyrosinase (Figure 3). Extracts obtained from the parent plant exhibited concentration-dependent inhibitory activity, ranging from 13.91% to 62.89%. The highest inhibition was observed at a concentration of 0.2 mg/mL and reached 59.42% for the leaf extract and 62.89% for the herb extract. The calculated IC_50_ values were 0.17 ± 0.01 mg/mL for the leaf extract and 0.16 ± 0.02 mg/mL for the herb extract, indicating comparable tyrosinase inhibitory activity.

The inhibitory activity of microshoot culture extracts ranged from 12.17% to 31.32%, depending on the extract concentration and cultivation period (Figure 3). The highest inhibition was observed at a concentration of 0.2 mg/mL. At this concentration, the inhibition values ranged from 24.59% for the extract obtained after 30 days of cultivation to 31.32% for the extract obtained after 10 days. As none of the microshoot culture extracts reached 50% inhibition within the tested concentration range, their IC_50_ values could not be reliably determined.

#### 2.3.3. Elastase Inhibitory Assay

**Figure 4:**
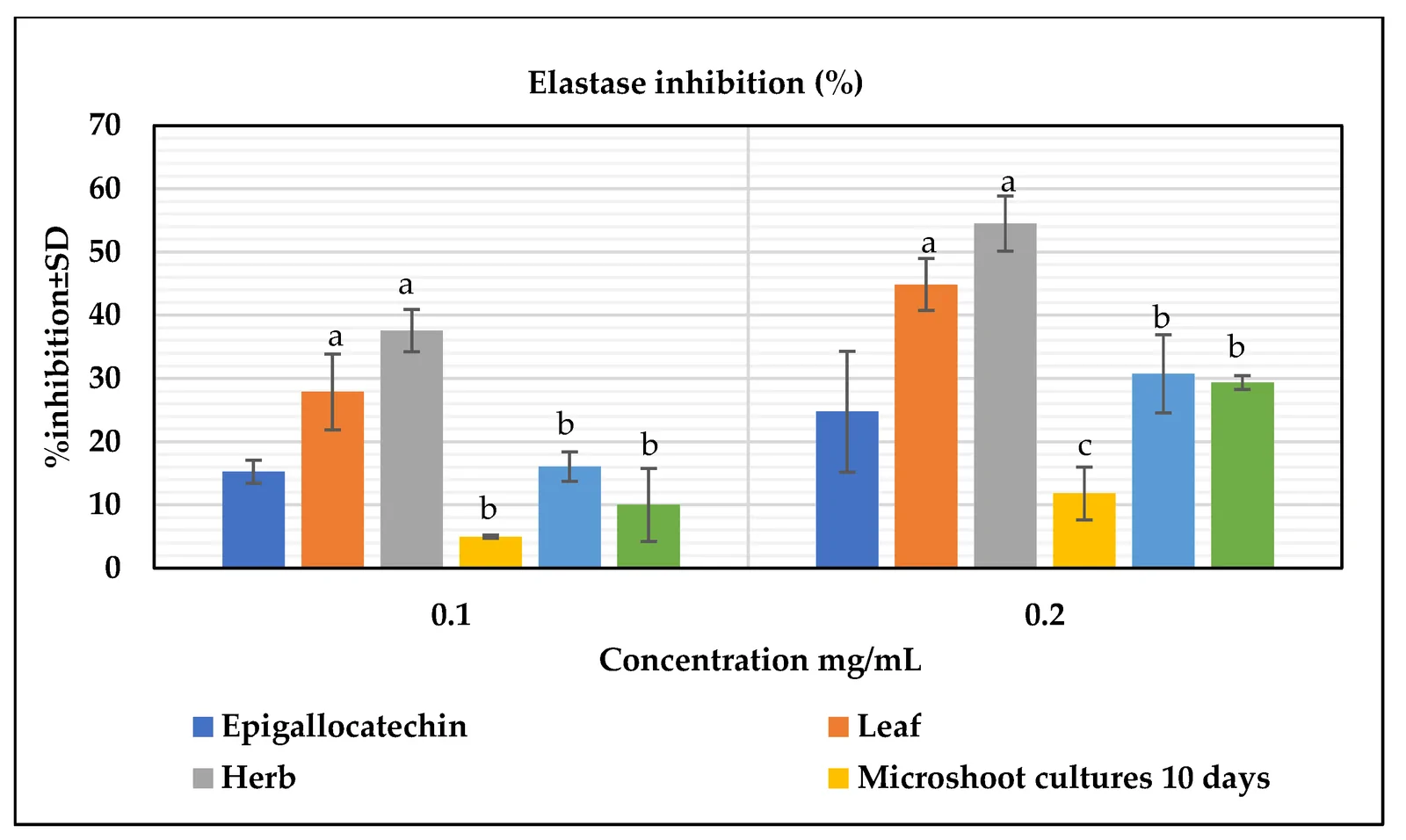
Inhibitory activity of leaf, herb, and microshoot cultures of *L. germanicus* extracts on elastase, in comparison with epigallocatechin. Data are presented as mean ± SD of three independent biological replicates (*n* = 3); different lowercase letters indicate statistically significant differences between samples at the same concentration (*p* < 0.05).

The elastase inhibitory activity of *L. germanicus* extracts was evaluated at concentrations of 0.1 and 0.2 mg/mL (Figure 4). Extracts obtained from the parent plant exhibited the strongest inhibitory activity, ranging from 27.87% to 54.49%, depending on the extract type and concentration. The highest inhibition was observed for the herb extract. For the leaf extract, 50% inhibition was not achieved within the tested concentration range; therefore, its IC_50_ value could not be reliably determined.

For the microshoot culture extracts, elastase inhibition ranged from 4.99% to 30.74%, depending on the extract concentration and cultivation period (Figure 4). The highest inhibition was recorded for the extract obtained from microshoot cultures after 20 days of cultivation. A comparable inhibition value of 30.00% was observed for the extract obtained after 30 days. As none of the microshoot culture extracts reached 50% inhibition within the tested concentration range, their IC_50_ values could not be reliably determined.

#### 2.3.4. Anti-Microbial Assay

The extracts of *L. germanicus* exhibited selective antimicrobial activity, predominantly against Gram-positive bacteria (Table 2). The strongest effect was observed against *S. aureus*, with MIC (minimum inhibitory concentration) values of 5 mg/mL for the 30-day microshoot culture extract and 10 mg/mL for the 20-day microshoot culture extract. The 20-day microshoot culture extract also inhibited *S. agalactiae* and *S. epidermidis* at an MIC of 20 mg/mL. Activity against *S. mutans* was observed for the herb extract as well as for extracts obtained from 10-, 20-, and 30-day microshoot cultures, all with an MIC of 20 mg/mL. In contrast, the leaf extract showed no detectable antibacterial activity within the tested concentration range. None of the extracts inhibited *C. albicans*, *E. coli*, or *P. aeruginosa* at concentrations up to 20 mg/mL. Overall, the antimicrobial activity was mainly restricted to Gram-positive cocci, whereas Gram-negative rods and *C. albicans* showed markedly lower susceptibility under the applied experimental conditions.

### 2.4. Principal Component and Hierarchical Clustering Analyses

**Figure 5:**
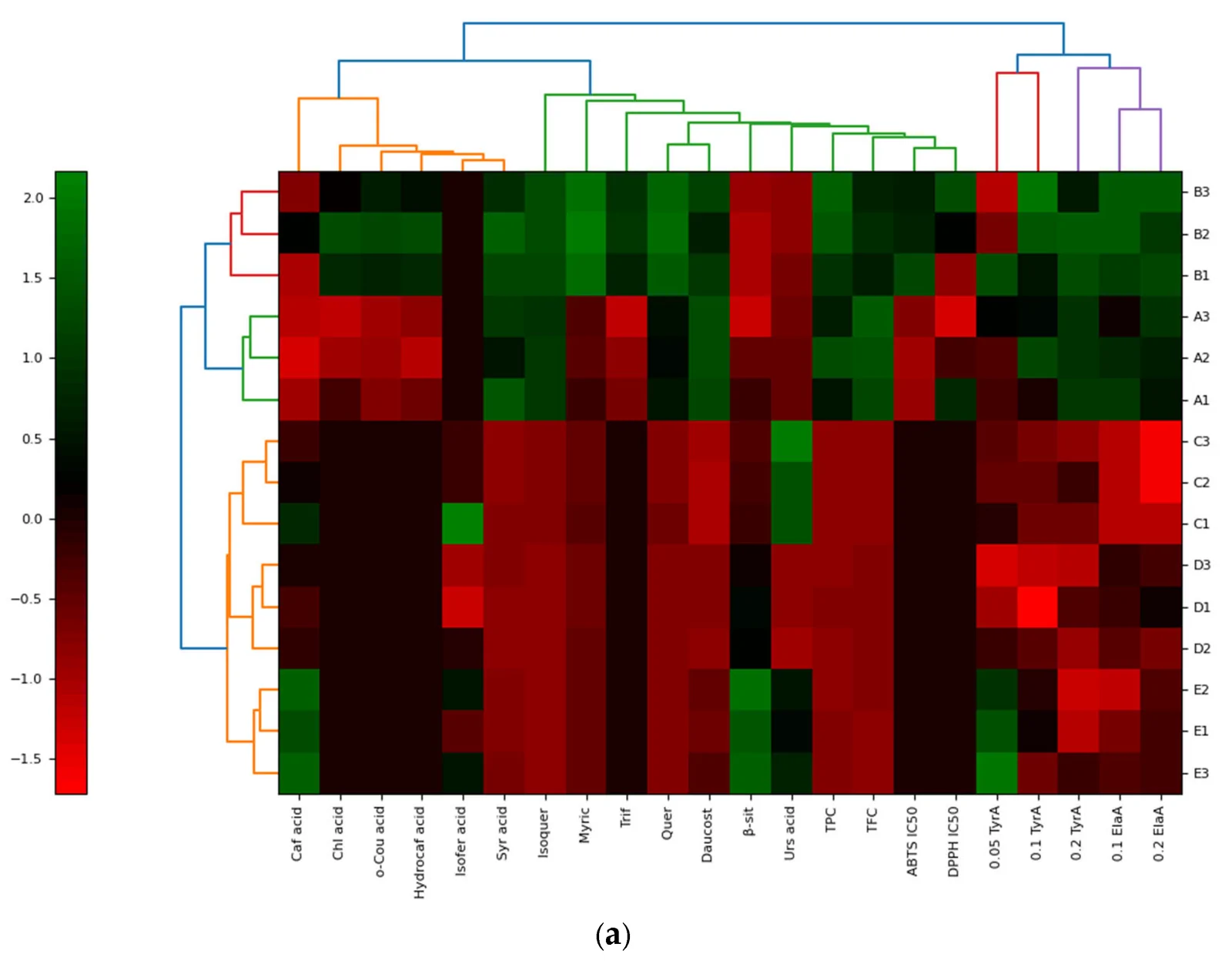

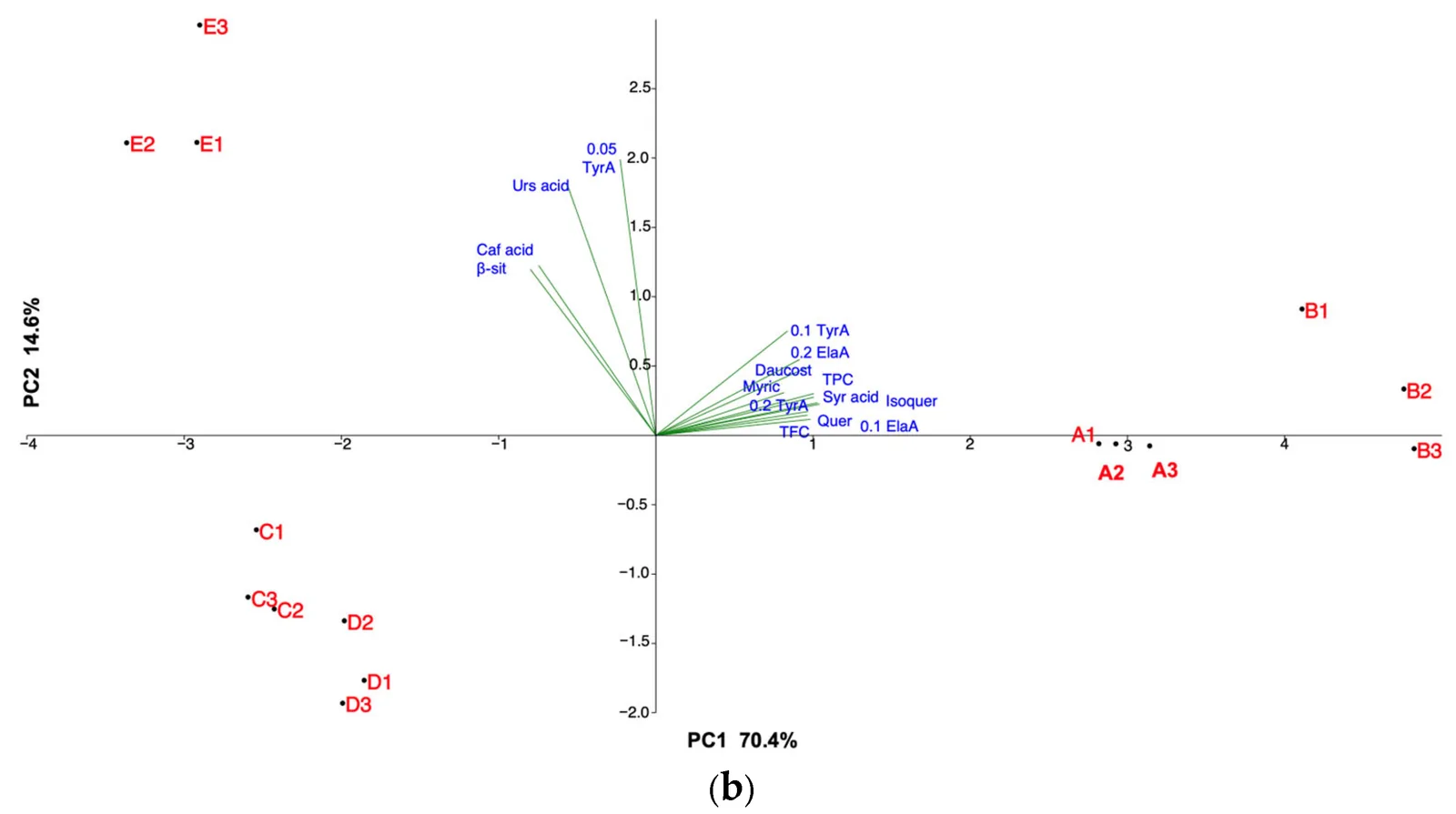
Phytochemical and biological profiles of *L. germanicus* leaf, herb, and microshoot culture extracts harvested after 10, 20, and 30 days. (**a**) Heatmap with hierarchical clustering of individual biological replicates, with each variable standardized column-wise using z-scores; Green and red indicate values above and below the respective variable mean, respectively; (**b**) PCA biplot based on 15 variables with complete numerical data across 15 individual biological replicates, with each variable standardized using z-scores. PC1 and PC2 explain 70.4% and 14.6% of the total variance, respectively. Sample codes in both panels: A1–A3, leaf extracts; B1–B3, herb extracts; C1–C3, 10-day microshoot culture extracts; D1–D3, 20-day microshoot culture extracts; and E1–E3, 30-day microshoot culture extracts. Green line in panel (**b**) represent variable loading vectors.

The heatmap and PCA of individual biological replicates showed a consistent distinction between parent-plant and microshoot culture extracts (Figure 5). Hierarchical clustering grouped the leaf and herb samples into separate subclusters within a common parent-plant branch, whereas the microshoot culture samples formed a separate main branch. Replicates from each cultivation period also clustered together. Parent-plant extracts generally showed higher contents of several phenolic constituents, TPC, and TFC, together with stronger tyrosinase inhibition at 0.1 and 0.2 mg/mL and elastase inhibition at both tested concentrations. PCA based on 15 variables with complete numerical data across 15 biological replicates supported this overall pattern. PC1 explained 70.4% of the total variance and separated the parent-plant samples from the culture samples, while PC2 explained 14.6% and distinguished the 30-day culture samples from the 10- and 20-day samples. Together, the first two components accounted for 85.0% of the total variance. The 30-day culture extracts had the highest mean caffeic acid, β-sitosterol, and ursolic acid contents, as well as the strongest mean tyrosinase inhibition at 0.05 mg/mL, consistent with their position in the biplot. Individual replicates generally occupied similar regions within each experimental group, while also revealing within-group variability.

**Figure 6:**
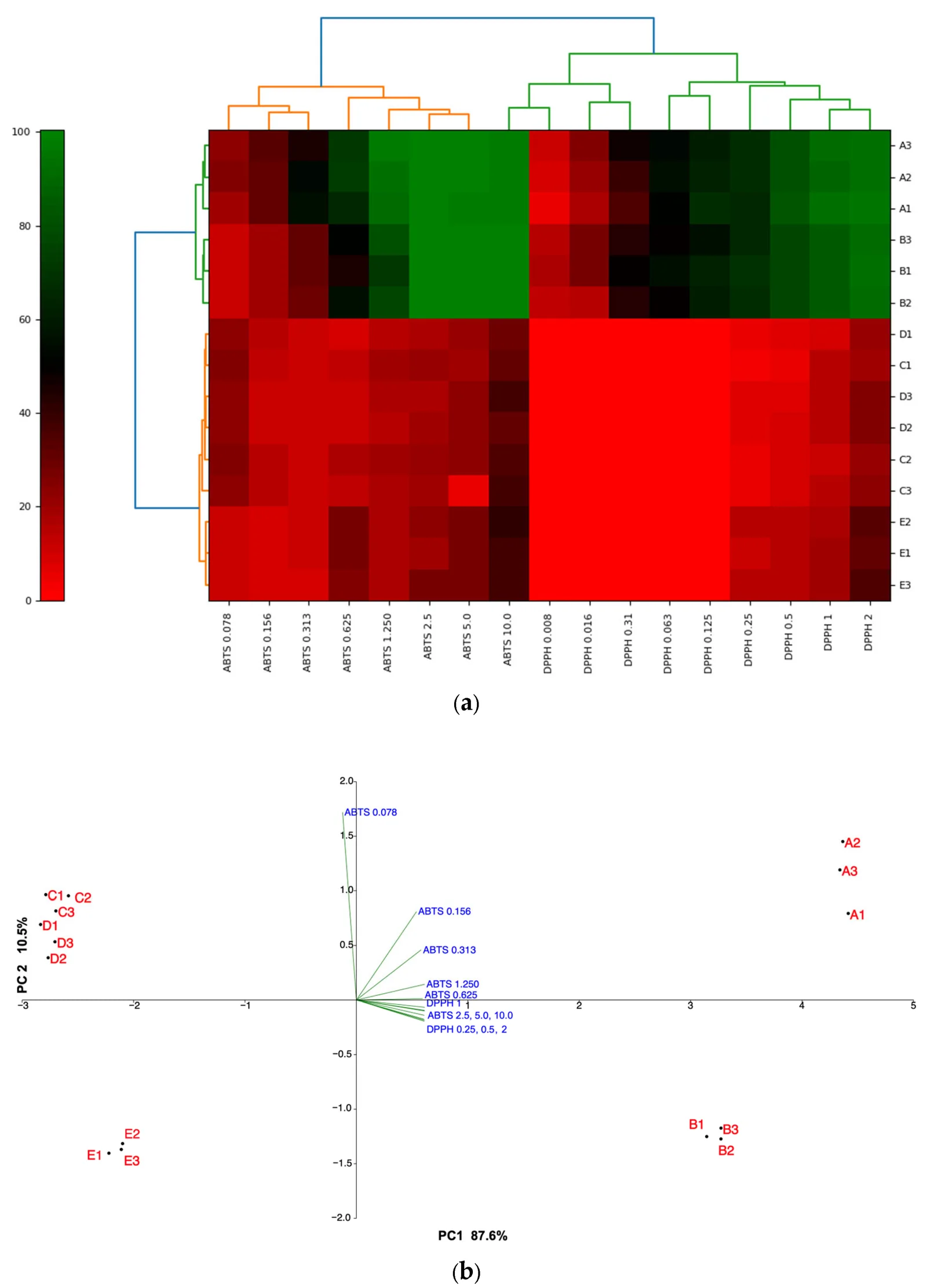
ABTS and DPPH radical-scavenging profiles of *L. germanicus* leaf, herb, and microshoot culture extracts harvested after 10, 20, and 30 days. (**a**) Heatmap with hierarchical clustering of individual biological replicates, displaying responses at eight ABTS and nine DPPH concentrations (17 variables). The heatmap colour scale represents radical inhibition (%), with green indicating higher and red indicating lower inhibition; (**b**) PCA biplot based on eight ABTS and four DPPH variables with complete numerical data across all 15 biological replicates. Each PCA variable was independently standardized using z-scores across all 15 samples. The four DPPH concentrations included in PCA were 0.25, 0.5, 1, and 2 mg/mL. PC1 and PC2 explain 87.6% and 10.5% of the total variance, respectively. Sample codes in both panels: A1–A3, leaf extracts; B1–B3, herb extracts; C1–C3, 10-day microshoot culture extracts; D1–D3, 20-day microshoot culture extracts; and E1–E3, 30-day microshoot culture extracts. Green line in panel (**b**) represent variable loading vectors; closely aligned vectors are labelled jointly for readability. Numbers accompanying ABTS and DPPH indicate extract concentrations (mg/mL).

The combined ABTS–DPPH heatmap and PCA of individual biological replicates distinguished the parent-plant extracts from the microshoot culture extracts (Figure 6). Hierarchical clustering grouped leaf and herb samples within a common parent-plant branch, separate from the culture samples. The 10- and 20-day culture samples were intermingled within the culture branch, whereas the 30-day samples formed a distinct subgroup. Parent-plant extracts generally showed higher radical-scavenging responses at ABTS concentrations of 0.156–10 mg/mL and at all four DPPH concentrations with numerical results for every sample. Among cultures, the 30-day extracts showed the highest mean DPPH inhibition at all shared concentrations and higher mean ABTS responses at several concentrations. The lowest ABTS concentration, 0.078 mg/mL, showed a different ranking, with the 10- and 20-day cultures giving mean responses comparable to the leaf extracts. PCA of eight ABTS and four DPPH variables measured across 15 biological replicates supported the overall distinction. PC1 explained 87.6% of the total variance and separated the parent-plant samples from the culture samples, whereas PC2 explained 10.5%, bringing the cumulative explained variance to 98.1%. PC2 distinguished leaf samples from herb samples and the 30-day culture samples from the 10- and 20-day samples. The loading vector for ABTS at 0.078 mg/mL was oriented predominantly along positive PC2, while most other variable vectors were oriented primarily along positive PC1. Replicates from each experimental group occupied similar regions of the biplot, while also revealing within-group variability.

## 3. Discussion

This study presents the first comprehensive characterization of the phytochemical composition and biological potential of *L. germanicus*. The research focused on the occurrence and accumulation of polyphenolic compounds, sterols and triterpenoids as well as the assessment of antioxidant activity and inhibitory effects against elastase and tyrosinase. Extracts obtained from leaf, herb and microshoot cultures of *L. germanicus* were analyzed.

To date, no comprehensive studies have addressed the chemical composition and biological activity of *L. germanicus*, and no in vitro cultures have previously been established or investigated for species belonging to the *Lotus* sect. *Dorycnium* (formerly genus *Dorycnium*). Therefore, the present work represents the first comprehensive investigation of *L. germanicus* at both the phytochemical and biological levels and, to the best of our knowledge, the first report on the establishment and characterization of in vitro cultures within section *Dorycnium*. The successful establishment of *L. germanicus* microshoot cultures thus provides a novel biotechnological platform for investigating the biosynthetic potential of this poorly studied species. Biotechnological approaches play an important role in the conservation and sustainable utilization of rare and insufficiently investigated plant species, as they enable the production of plant biomass under controlled conditions and facilitate the evaluation of their chemical profiles and biological potential. Numerous studies have demonstrated that rare, endemic, and threatened species can be successfully propagated under in vitro conditions and may accumulate valuable secondary metabolites or exhibit biological activities comparable to, and in some cases exceeding, those of parent plants [24,25]. For example, Türker et al. [26], studying the endemic species *Hypericum bilgehan-bilgilii* Basköse & Savran, reported improved physiological performance and increased levels of antioxidant compounds in plants cultivated in vitro. These findings emphasize the importance of plant tissue culture as a valuable biotechnological strategy for the conservation, propagation, and exploration of bioactive compounds from poorly studied plant species.

The phytochemical analysis of *L. germanicus* revealed generally similar qualitative and quantitative profiles in the herb and leaf extracts, with no pronounced differences in the accumulation of the major identified compounds. Among the flavonoids, isoquercitrin and trifolin were the predominant constituents in both extracts, with higher amounts generally observed in the herb. A similar pattern was noted for phenolic acids, with hydrocaffeic acid being the dominant compound in both the herb and leaf extracts and occurring at a higher level in the herb. In contrast, marked differences were observed between the parent plant and the in vitro microshoot cultures. The microshoot cultures did not contain all compounds detected in the parent plant, and the amounts of most identified constituents were considerably lower. The accumulation of metabolites in the in vitro cultures was also dependent on the duration of cultivation. The highest contents of phenolic acids and flavonoids were generally observed after 10 days of culture, whereas 30-day-old microshoot cultures were characterized by higher levels of sterol-related compounds and ursolic acid. These findings indicate that the duration of the growth cycle may influence the biosynthetic capacity of *L. germanicus* microshoot cultures and could therefore be an important factor in the optimization of secondary metabolite production. Future research should therefore focus on optimization of culture conditions and the application of strategies aimed at stimulating secondary metabolite biosynthesis, including modification of the growth regulator composition, optimization of the culture duration, elicitation, precursor feeding, or other approaches commonly used to enhance metabolite production in plant in vitro systems.

The phytochemical profile of *L. germanicus* confirmed the presence of several groups of secondary metabolites, particularly flavonoids and phenolic acids. Previous investigations of the former genus *Dorycnium* have mainly focused on total phenolic and flavonoid contents. Stefanović et al. [16] reported a total phenolic content of 7577 mg GAE/100 g extract and a total flavonoid content of 11,007 mg RE/100 g extract in ethanolic extracts of *D. herbaceum*. In the present study, the corresponding values determined for the herb of *L. germanicus* were 260.98 mg GAE/100 g DW and 723.61 mg RE/100 g DW, respectively. *Dorycnium herbaceum* was also investigated by Nicolaescu et al. [19], who reported total phenolic and total flavonoid contents of 0.732 mg GAE/L and 0.495 mg QE/L, respectively. Nevertheless, direct quantitative comparison between these studies is limited by differences in extraction procedures, reference standards, analytical conditions, and the units used to express the results. In the present study, the concentrations of individual flavonoids determined by HPLC-DAD were considerably higher than the spectrophotometrically estimated TFC values, particularly for isoquercitrin. This discrepancy may be partly attributed to differences in the analytical principles and sample preparation procedures. HPLC-DAD was performed directly on ethanolic extracts, with individual compounds quantified using authentic reference standards, whereas the TFC assay was conducted on reconstituted dry extracts and expressed as rutin equivalents. Furthermore, the aluminum chloride colorimetric assay is influenced by the structural characteristics and complexation behavior of individual flavonoids, as previously discussed by Shraim et al. [27]. Consequently, TFC values expressed as rutin equivalents should not be interpreted as directly equivalent to the sum of individual flavonoids quantified by HPLC-DAD. Nevertheless, the substantial discrepancy between these measurements warrants cautious interpretation. Only a few studies have addressed the composition of individual phytochemicals in *Dorycnium* species. Moreno et al. [10] identified kaempferol derivatives, including kaempferol-3,7-*O*-α-di-rhamnopyranoside and kaempferol-3-*O*-β-glucopyranoside-7-*O*-α-rhamnopyranoside, in *D. rectum*. In contrast, neither kaempferol nor its derivatives were detected in the parent plant or in vitro cultures of *L. germanicus* analyzed in the present study. Movsumov et al. demonstrated the presence of myricetin, myricitrin, kaempferol, and quercetin in extracts from the aerial parts of *D. graecum*. Among these compounds, myricetin and quercetin were also detected in *L. germanicus*, together with isoquercitrin and trifolin, whereas kaempferol and myricitrin were not identified. These differences suggest considerable interspecific variability in the flavonoid composition of the genus. Movsumov et al. [12] also reported β-sitosterol and ursolic acid in *D. graecum*. Ursolic acid was likewise detected in *L. germanicus*, while daucosterol was additionally identified in the present study. Several phenolic acids were identified and quantified in *L. germanicus*, with hydrocaffeic acid representing the predominant compound within this group. Overall, these findings broaden the current knowledge of the phytochemical composition of *L. germanicus* and indicate distinct interspecific differences within the genus *Dorycnium*.

The ability of in vitro cultures to synthesize polyphenolic compounds has been demonstrated in other representatives of the Fabaceae family. For example, callus cultures of *Astragalus onobrychis* were shown to produce a diverse range of flavonoids, including derivatives of quercetin, luteolin, naringenin, apigenin, and kaempferol [28]. Similarly, in vitro shoot cultures of *Pterospartum tridentatum* retained the ability to synthesize phenolic compounds, although their total phenolic acid content was lower than that observed in the parent plant [29]. Moreover, not all phenolic acids and flavonoids identified in the parent plant were detected in the corresponding in vitro cultures, demonstrating qualitative as well as quantitative differences between field-grown material and cultured biomass. Quantitative differences in flavonoid accumulation have also been reported in in vitro cultures of *Cyclopia* species [30]. Importantly, the levels of individual flavonoids varied depending on the type of culture, with distinct accumulation patterns observed between callus and shoot cultures. These findings indicate that not only the transfer of plant material to in vitro conditions, but also the specific morphogenic pathway and type of cultured tissue may substantially influence secondary metabolite biosynthesis and accumulation. Comparable observations have also been reported for callus cultures of *Trifolium rubens*, in which distinct qualitative and quantitative differences in the phytochemical profiles of callus cultures and the parent plant were observed [31]. A similar pattern was found in the present study for *L. germanicus*. The microshoot cultures retained the capacity to synthesize several phenolic acids, flavonoids, sterol-related compounds, and ursolic acid; however, they did not reproduce the complete phytochemical profile of the parent plant, and the concentrations of most identified compounds were lower. These findings are consistent with observations for other Fabaceae species and indicate that transfer to in vitro conditions may substantially modify secondary metabolite biosynthesis. Such differences may result from changes in developmental stage, growth conditions, nutrient availability, plant growth regulator composition, and the absence of environmental stimuli normally experienced by plants growing under natural conditions.

Both antioxidant assays demonstrated that the parent-plant extracts of *L. germanicus* exhibited the highest antioxidant potential among the analyzed samples. However, the magnitude of the differences between the leaf and herb extracts varied depending on the assay used. In the DPPH method, the differences between these two extracts were less pronounced than in the ABTS assay, indicating an assay-dependent response. Such variation may result from differences in the phytochemical composition of the extracts as well as from the distinct reaction mechanisms underlying the ABTS and DPPH methods. The ABTS assay is responsive to a broad range of antioxidant compounds, including both hydrophilic and lipophilic constituents, whereas the DPPH method is more selective toward compounds capable of efficiently donating hydrogen atoms or electrons. Therefore, differences in the relative contribution of phenolic acids, flavonoids, and other antioxidant constituents may explain the variation observed between the two assays [32]. The extracts obtained from microshoot cultures also exhibited measurable antioxidant activity, which was influenced by the duration of cultivation. In both assays, the 30-day-old cultures generally showed the highest radical-scavenging activity among the microshoot extracts at most of the tested concentrations. Nevertheless, their antioxidant potential remained markedly lower than that of the parent-plant extracts. The maximum inhibition observed for the microshoot culture extracts was 37.75% in the ABTS assay at 10 mg/mL and 33.00% in the DPPH assay at 2 mg/mL, both recorded for the 30-day-old cultures. As none of the microshoot extracts achieved 50% radical inhibition within the tested concentration ranges, IC_50_ values could not be determined. The higher antioxidant activity of the parent-plant extracts may be associated with their substantially higher content of polyphenolic compounds, particularly phenolic acids and flavonoids, which are well known for their ability to scavenge free radicals and participate in redox reactions. This relationship is consistent with the phytochemical results obtained in the present study, which demonstrated considerably higher levels and greater diversity of phenolic constituents in the parent plant compared with the microshoot cultures. These findings indicate that *L. germanicus* microshoot cultures are capable of producing metabolites with antioxidant properties, although their overall radical-scavenging capacity is lower than that of the parent plant. The antioxidant properties of plant extracts are generally associated with the presence of diverse secondary metabolites, particularly phenolic acids and flavonoids. As discussed by Bashir et al. [33], plant-derived antioxidants may contribute to oxidative stress regulation through several mechanisms, including free radical scavenging and modulation of cellular antioxidant defenses. In this context, Alharthi et al. [34] reported protective effects of lotusine, an alkaloid derived from *Nelumbo nucifera*, against hyperglycemia-induced oxidative stress. However, this study involved a different plant species, bioactive compound, and experimental model, and its findings cannot be directly extrapolated to *L. germanicus*. Importantly, the ABTS and DPPH assays used in the present study provide information on chemical radical-scavenging capacity rather than direct evidence of protection against oxidative stress in biological systems. Therefore, further investigations using cellular models are needed to establish the biological relevance of the antioxidant activity observed for *L. germanicus* extracts. To date, there are no available studies concerning the antioxidant potential of *L. germanicus*. However, several reports have evaluated the antioxidant activity of other species belonging to the genus *Dorycnium*. Stefanović et al. [16] investigated the DPPH radical-scavenging activity of *D. herbaceum* extracts obtained using different solvents and demonstrated that the ethanolic extract exhibited the lowest EC_50_ value, defined as the effective concentration required to scavenge 50% of DPPH radicals, compared with the acetone and ethyl acetate extracts. The EC_50_ value of the ethanolic *D. herbaceum* extract was 0.084 mg/mL. In the present study, lower DPPH IC_50_ values were obtained for *L. germanicus*, reaching 0.04 mg/mL for the leaf extract and 0.05 mg/mL for the herb extract. Although direct comparison should be interpreted with some caution due to possible differences in extraction procedures and assay conditions, these results suggest a strong antioxidant potential of *L. germanicus* and indicate that its parent-plant extracts may be at least comparable to, or more active than, those previously reported for *D. herbaceum*. Antioxidant properties of another representative of the genus were demonstrated by Pistelli et al. [8], who evaluated ethyl acetate and n-butanol extracts obtained from the aerial parts of *D. hirsutum* using the DPPH assay. The ethyl acetate extract showed higher antioxidant activity, reaching 0.60 mol TE/g, whereas the activity of the n-butanol extract was approximately twofold lower (0.29 mol TE/g). Moreover, flavonol glycosides isolated from *D. hirsutum*, including derivatives of kaempferol and quercetin, exhibited pronounced radical-scavenging activity, with values ranging from 0.39 to 1.23 mol TE/g. These findings support the important contribution of flavonoids to the antioxidant properties of *Dorycnium* species. Antioxidant activity has also been investigated in other representatives of the Fabaceae family. Ethanolic extracts obtained from the aerial parts, including stems and leaves, of *Lotus creticus* were evaluated using both the ABTS and DPPH assays. The IC_50_ values reported for *L. creticus* were higher than those obtained for *L. germanicus* in the present study. Specifically, the IC_50_ value in the ABTS assay was approximately 2.9-fold higher, while that determined using the DPPH method was approximately 22.8-fold higher than the corresponding value obtained for *L. germanicus* [35]. Since lower IC_50_ values indicate greater radical-scavenging activity under comparable assay conditions, these results suggest that *L. germanicus* extracts may exhibit higher antioxidant activity than *L. creticus* extracts under the respective experimental conditions, particularly in the DPPH assay. However, direct comparison should again be approached with caution because differences in plant material, extraction procedures, solvent composition, and assay conditions may substantially affect the obtained IC_50_ values. The pronounced antioxidant activity of the parent-plant extracts of *L. germanicus* may be associated with their high content and diversity of polyphenolic compounds, particularly phenolic acids and flavonoids. In the present study, quercetin and its derivatives, including isoquercitrin, as well as trifolin and several phenolic acids were identified, with hydrocaffeic acid being one of the predominant constituents. These compounds may therefore contribute substantially to the radical-scavenging activity observed in both the ABTS and DPPH assays. Similar findings have been reported for *T. rubens*, where callus cultures exhibited lower radical-scavenging activity than the parent plant [31], supporting the association between altered phenolic profiles under in vitro conditions and reduced antioxidant capacity. The agreement between the heatmap and PCA further supported the overall differences in radical-scavenging profiles between the parent-plant and in vitro-derived extracts. Parent-plant extracts generally exhibited stronger responses across both antioxidant assays. Among the microshoot cultures, the 30-day extract showed higher DPPH activity, suggesting that cultivation duration may influence antioxidant potential. However, the ABTS results did not follow the same ranking at all concentrations, including the low-concentration exception, indicating that the observed differences cannot be interpreted as a uniform activity gradient.

The tyrosinase inhibitory activity of *L. germanicus* extracts was dependent on both extract origin and concentration. Extracts obtained from the parent plant, particularly the leaf and herb extracts, inhibited mushroom tyrosinase activity in a concentration-dependent manner, with the highest inhibition reaching 62.89% at 0.2 mg/mL. Extracts from in vitro microshoot cultures also exhibited tyrosinase inhibitory activity, although their effect was considerably weaker. The highest inhibition observed for the microshoot cultures was 31.32% at 0.2 mg/mL, indicating approximately twofold lower activity compared with the most active parent-plant extract. These differences may be related to the lower content and reduced diversity of phenolic compounds in the in vitro cultures, particularly flavonoids that may contribute to tyrosinase inhibition. To date, information on the tyrosinase inhibitory activity of *Dorycnium* species remains limited. Placines et al. [35] reported tyrosinase inhibitory activity for *L. creticus*, with an activity of 27.84 mg kojic acid equivalents/g extract. Although the results cannot be directly compared with those obtained in the present study because of the different approaches used to express tyrosinase inhibition, they further indicate that representatives of this group of Fabaceae may constitute a source of compounds capable of modulating tyrosinase activity. The observed activity of *L. germanicus* may be associated, at least partly, with its flavonoid composition. Lou et al. [36] demonstrated that compounds isolated from the leaves of the Fabaceae species *Cercis glabra*, including quercetin and myricetin, exhibited pronounced tyrosinase inhibitory effects. Quercetin inhibited tyrosinase activity by 77.29%, with an IC_50_ value of 0.64 mM, whereas myricetin produced approximately 60% inhibition and an IC_50_ value of 1.39 mM. Both quercetin and myricetin were also identified in *L. germanicus* in the present study, suggesting that these compounds may contribute to the observed tyrosinase inhibitory activity, although possible synergistic effects of other phenolic constituents should also be considered. Recent findings also support a potential contribution of ursolic acid to the tyrosinase inhibitory activity of *L. germanicus*. Maisto et al. [37] demonstrated that pure ursolic acid inhibited mushroom tyrosinase, with an IC_50_ value of 26.82 µg/mL. Since ursolic acid was identified in both the parent plant and in vitro microshoot cultures of *L. germanicus* in the present study, this compound may partly contribute to the tyrosinase inhibitory activity observed in both types of extracts. Its presence, together with quercetin, myricetin, and other phenolic constituents, may therefore contribute to the overall anti-tyrosinase potential of *L. germanicus*. Differences between parent plants and in vitro cultures have also been reported for another representative of the Fabaceae family. In previous studies on *T. rubens*, callus culture extracts did not produce a measurable reduction in mushroom tyrosinase activity, whereas flower extracts of the parent plant inhibited the enzyme by approximately 25% at 0.1 mg/mL [31]. This activity was lower than that observed for *L. germanicus* in the present study. Taken together, these findings indicate that *L. germanicus*, particularly extracts obtained from the parent plant, possesses promising tyrosinase inhibitory potential, while its microshoot cultures retain this biological activity to a lesser extent. The differences between the parent plant and in vitro cultures further support the importance of secondary metabolite composition and accumulation for the biological properties of the extracts.

The elastase inhibitory activity of *L. germanicus* extracts varied depending on the origin of the plant material and the duration of in vitro culture. The highest activity was observed for the herb extract, which inhibited elastase by 54.95% at a concentration of 0.2 mg/mL. Extracts obtained from microshoot cultures also demonstrated elastase inhibitory activity, with the strongest effect recorded for cultures harvested after 20 days. However, this activity was approximately 1.79-fold lower than that observed for the herb extract, indicating that the parent plant possessed a considerably stronger anti-elastase potential. Anti-elastase activity has also been reported for other representatives of the Fabaceae family. Extracts from *Mucuna gigantea*, *M. interrupta*, *M. monosperma*, and *M. pruriens* inhibited elastase activity within the range of 32.12–39.48%, confirming the anti-aging potential of species belonging to this family [38]. A particularly strong effect was reported for *Spatholobus suberectus*, whose extract inhibited elastase activity by approximately 66% at a concentration of only 0.05 mg/mL [39]. *Lotus maritimus* has likewise been reported to exhibit moderate elastase inhibitory activity, further supporting the occurrence of this biological property among Fabaceae species [40]. Comparable observations have also been reported for *T. rubens*. Extracts obtained from the flowers of the parent plant inhibited elastase activity by up to approximately 35%, whereas callus culture extracts showed slightly lower activity, reaching approximately 29% [31]. A similar tendency was observed in the present study, where *L. germanicus* microshoot cultures retained the ability to inhibit elastase, although their activity was lower than that of the parent plant. These findings may reflect qualitative and quantitative differences in the secondary metabolite profiles of naturally grown plants and in vitro cultures. In particular, the higher content and greater diversity of phenolic acids and flavonoids in the parent plant may contribute to its stronger elastase inhibitory activity. In addition, ursolic acid, which was detected in both the parent plant and microshoot cultures of *L. germanicus*, may also contribute to the observed effect. Feng et al. [41] demonstrated that ursolic acid possesses potent inhibitory activity against human neutrophil elastase, with an IC_50_ value of 5.51 µM, and produced more than 80% inhibition at 30 µM. The combined heatmap and PCA further highlighted differences between the parent-plant and microshoot culture extracts across multiple phytochemical and biological variables. Higher contents of several phenolic constituents in the parent-plant extracts coincided with stronger inhibition in several enzyme assays, although these associations do not establish a causal contribution of individual compounds. Notably, the 30-day microshoot culture showed a distinct profile, characterized by higher caffeic acid, β-sitosterol, and ursolic acid contents and stronger tyrosinase inhibition at the lowest tested concentration. This pattern further suggests that cultivation duration may influence both metabolite accumulation and biological activity, although such changes do not uniformly enhance all investigated endpoints.

The antimicrobial activity of *L. germanicus* showed a clear preference toward Gram-positive bacteria, particularly *S. aureus*, whereas the tested Gram-negative bacteria and *C. albicans* were not susceptible within the examined concentration range. The MIC values of 5–20 mg/mL indicate measurable but relatively limited antibacterial activity of the crude extracts. These findings are generally consistent with previous reports demonstrating antimicrobial properties in related *Dorycnium* species, although the susceptibility patterns vary considerably among species and extracts. Stefanović et al. [16] reported that *D. herbaceum* extracts exhibited activity against both Gram-positive and Gram-negative bacteria, with *Bacillus subtilis*, *S. aureus*, *P. aeruginosa*, and *Proteus mirabilis* among the most susceptible microorganisms, with MIC values ranging from 1.25 to 10 mg/mL. Antifungal activity was weaker, with detectable MIC values of 10–20 mg/mL. Similarly, Usta et al. [14] demonstrated antibacterial activity of *D. pentaphyllum* extracts; particularly, ethanolic and methanolic extracts showed inhibitory effects against selected Gram-negative bacteria. These observations indicate that antimicrobial activity within this group is not restricted exclusively to Gram-positive microorganisms and may depend strongly on the plant species, solvent used for extraction, and the chemical composition of the resulting extract. The potential contribution of polyphenolic constituents to this activity is also supported by studies on *D. rectum*. Sivakumaran et al. [11] demonstrated that proanthocyanidin fractions isolated from *D. rectum* inhibited several bacterial species and that, for some microorganisms, antibacterial activity depended on the structural characteristics of the proanthocyanidin fractions. This suggests that antimicrobial effects may be determined not only by the total amount of phenolic compounds but also by their qualitative profile and structural features. This interpretation may be particularly relevant to the present study, since the lowest MIC value (5 mg/mL) against *S. aureus* was recorded for the 30-day microshoot culture extract, despite the generally lower total phytochemical content of the in vitro-derived material compared with the parent plant. Thus, the observed activity may reflect differences in the relative proportions and combined effects of individual bioactive constituents rather than their total concentration alone. Nevertheless, direct comparison with previous studies should be made cautiously because of differences in plant species, extraction procedures, microbial strains, and antimicrobial assay methods. Recent studies have further highlighted the antimicrobial potential of plant-derived extracts and the importance of methodological considerations when evaluating their activity. Rodjan et al. [42] investigated the antifungal properties of *Piper betle* extracts and selected phenolic constituents against *Candida* species using broth microdilution assays, demonstrating species-dependent susceptibility. Similarly, Ullah et al. [43] reported antimicrobial properties of *Lawsonia inermis* extracts obtained from leaves and stems. Although these studies involved different plant species, microbial targets, and experimental conditions, they provide additional context for evaluating the antimicrobial potential of plant extracts. Importantly, differences in assay procedures, inoculum preparation, solvent concentrations, and endpoint determination should be considered when comparing antimicrobial activities across studies.

The agreement between the heatmap and PCA indicates that the tested parent-plant and culture extracts differed across multiple chemical and biological endpoints. Higher contents of several phenolic constituents in the parent-plant extracts coincided with stronger inhibition in several enzyme assays, although this pattern does not establish a causal contribution of individual compounds. The distinct 30-day culture profile, including higher caffeic acid, β-sitosterol, and ursolic acid contents and stronger tyrosinase inhibition at the lowest tested concentration, suggests that cultivation duration may influence extract composition and activity without improving all endpoints uniformly.

Although the phytochemical composition and biological activities of *L. germanicus* extracts suggest their potential relevance to cosmetic research, the present findings should be considered preliminary. The antioxidant, tyrosinase-inhibitory, elastase-inhibitory, and antibacterial activities were evaluated using in vitro assays, which do not directly establish efficacy under physiological skin conditions. Recent studies on polyphenol-rich plant extracts, including the investigation by Altarjami et al. [44] on *Punica granatum* peel extracts, have highlighted their diverse biological activities; however, such findings cannot be directly extrapolated to cosmetic efficacy. Moreover, the present study did not include skin-relevant cellular models, skin irritation assessments, or evaluations of formulated cosmetic products. Therefore, further investigations involving cytotoxicity testing, skin compatibility assessments, reconstructed human epidermis models, and appropriately formulated products are necessary to confirm the safety and biological efficacy of *L. germanicus* extracts for potential cosmetic applications.

## 4. Materials and Methods

### 4.1. Parent-Plant Material

Leaf, herb as well as the seeds of *L. germanicus* (Figure 1a) were collected in August 2025 from the Botanical Garden in Kielce (50°51′59.20″ N, 20°35′56.33″ E, 280 m a.sl.). The *L. germanicus* seedlings were originally introduced into the garden’s collection of rare and endangered species from their natural habitat in the Małopolska Upland, Nida Basin, from Pińczowski Hump, between Pińczów and Skowronno in the Pińczów administrative district, where it grows on xerothermic grasslands. *Lotus germanicus* (syn. *Dorycnium germanicum*) is a critically rare species in Poland, reaching its northern distribution limit here. It is strictly protected by national law [45] and listed as Endangered (EN) in both the Polish Red Data Book of Plants [46] and the national red list [7]. The seeds were obtained in accordance with the permit (no. WPN.I.6400.3.3.2021.MM) from the Regional Director for Environmental Protection in Kielce. This permit covered the original collection of seeds from the natural population; these seeds were subsequently sown, and the resulting seedlings were planted in the Botanical Garden in Kielce, where the leaves, herb, and seeds used in this study were collected from the cultivated plants. The plant species was identified by Prof. Renata Piwowarczyk. The seeds were stored at the Center for Research and Conservation of Biodiversity (Institute of Biology, Jan Kochanowski University, Uniwersytecka 7, 25-406 Kielce, Poland; 50.882032, 20.657719). Prior to analysis, the plant material was lyophilized using a Labconco freeze dryer (Labconco Corporation, Kansas City, MO, USA). Reference specimens were deposited in the Herbarium of Jan Kochanowski University in Kielce (KTC), located at the Institute of Biology, Uniwersytecka 7, 25-406 Kielce, Poland (50.882032, 20.657719). The voucher specimen documenting the material used in this study bears the unique identifier [KTC-LG-2021-01]. Species are organized alphabetically in boxes labeled with their respective species names, along with a label indicating the collection site and the material used for analysis.

### 4.2. Initiation of In Vitro Cultures

The seeds of *L. germanicus* were surface-sterilized with 0.1% (*v*/*v*) chloramine T (WARCHEM Sp. z o.o., Zakręt, Poland) for 10 min while being continuously stirred, followed by three rinses with sterile distilled water. Sterilized seeds were then inoculated onto Murashige and Skoog (MS) medium [47] supplemented with 0.8% (*w*/*v*) plant agar (Duchefa, The Netherlands), 3% (*w*/*v*) sucrose, 1 mg/L BA, 0.5 mg/L NAA, and 0.25 mg/L GA_3_ (pH = 5.8). After four weeks, a shoot was formed. The in vitro cultures were maintained at 25 ± 2 °C under continuous white artificial LED illumination with an intensity of 1 W/m^2^ (Heckermann, Poland) and subcultured every 20 days.

### 4.3. Experimental In Vitro Cultures

The experiment included the cultivation of *L. germanicus* microshoot on agar medium. Microshoot cultures were maintained on standard MS medium supplemented with 0.8% (*w*/*v*) plant agar (Duchefa, The Netherlands), 3% (*w*/*v*) sucrose, 1 mg/L BA, 0.5 mg/L NAA, and 0.25 mg/L GA_3_. The cultures were incubated under continuous white light (1 W/m^2^) at 25 ± 2 °C and harvested after 10, 20, and 30 days in three independent experimental series. For each cultivation period (10, 20, and 30 days), three independent experimental series were performed. In each experimental series, biomass collected from five separate culture vessels was pooled to obtain one biological replicate. Thus, three independent pooled biological replicates (*n* = 3) were obtained for each cultivation period. Each biological replicate was subsequently analyzed using technical replicate measurements, which were used to assess analytical variability and were not considered independent experimental units.

### 4.4. Extraction

Lyophilized (Labconco Corporation, Kansas City, MO, USA) and pulverized biomass from leaf, herb and microshoot cultures was extracted in 70% ethanol (*v*/*v*) (ChemLand, Piekary Śląskie, Poland). For each microshoot cultivation period, extraction was performed using the pooled lyophilized biomass collected from five culture vessels. The selection of 70% ethanol was based on its effectiveness in extracting a broad range of both polar and moderately non-polar bioactive compounds, including phenolics and flavonoids, while maintaining low toxicity and compatibility with biological assays. Extraction was performed using an ultrasonic bath (Sonic-6, Polsonic, Warsaw, Poland) (30 min, room temperature). The extracts were prepared at a concentration of 2% (*w*/*v*) by extracting 0.3 g of dried plant material with 15 mL of 70% ethanol. For biological assays 2% extracts were evaporated at 45 °C using a CentriVap vacuum concentrator (Labconco Corporation, Kansas City, MO, USA) and stored at −80 °C. For HPLC-DAD analysis, the obtained 2% extracts were filtered (Whatman No. 10 filter paper) and used for the determination of phytochemical compounds.

### 4.5. Total Phenolic Assay

TPC was determined in plant extracts using the Folin–Ciocalteu assay according to the protocol of Singleton and Rossi [48]. A 10 µL aliquot of 20 mg/mL ethanolic extract was combined with 10 µL 15% NaHCO_3_ (WARCHEM Sp. z o.o., Zakręt, Poland), 200 µL deionized water, and 20 µL FC reagent (Sigma-Aldrich, St. Louis, MO, USA), then incubated at room temperature for 2 h. Absorbance was recorded at 765 nm on SPECTROstar Nano microplate reader (BMG LABTECH GmbH, Ortenberg, Germany). Gallic acid (Sigma-Aldrich, St. Louis, MO, USA) solutions (0.0625, 0.125, 0.25, 0.5, 1, and 2 mg/mL) were used to prepare a calibration curve, and sample TPC values were calculated using the equation y = 3.0653x − 0.0313. The amounts of polyphenols were determined and expressed as GAE in mg/100 g DW.

### 4.6. Total Flavonoid Assay

TFC was estimated spectrophotometrically according to a method proposed by Christ & Müller (1960) [49] and modified by Shraim et al. [27] and Sari et al. [50]. Briefly, 50 μL of 5 mg/mL ethanolic extract was mixed with 100 μL ethanol, 20 μL 10% AlCl_3_. The plate was gently shaken five times each up and down and left to right in the horizontal plane. The plate was kept in incubation for three minutes. After the incubation, 20 μL 1M CH_3_COONa (WARCHEM Sp. z o.o., Zakręt, Poland) and 60 μL ethanol was added. The plate was incubated for 40 min in the darkness at room temperature and then absorbance was measured at 415 nm using a SPECTROstar Nano microplate reader (BMG LABTECH GmbH, Ortenberg, Germany). Rutin (Sigma-Aldrich, St. Louis, MO, USA) solution (0.0625, 0.125, 0.25, 0.5, 1, and 2 mg/mL) were used to prepare a calibration curve, and sample TFC values were calculated using the equation y = 2.8264x − 0.0046. The amounts of flavonoids were determined and expressed as RE in mg/100 g DW.

### 4.7. Polyphenol Profiling by HPLC-DAD

The determination of polyphenol concentration was carried out using the HPLC-DAD method, following the procedure outlined in earlier studies [51,52]. Ethanolic extracts were subjected to chromatographic analysis using an HPLC-DAD system (Merck-Hitachi, Merck KGaA, Darmstadt, Germany) equipped with a Purospher RP-18e analytical column (4 × 250 mm, 5 µm; Merck, Boston, MA, USA). Separation was performed with two mobile phases: phase A consisting of methanol and 0.5% acetic acid (1:4, *v*/*v*), and phase B composed of methanol. The gradient elution program was as follows: 0–20 min, 0% B; 20–35 min, 0–20% B; 35–45 min, 20–30% B; 45–55 min, 30–40% B; 55–60 min, 40–50% B; 60–65 min, 50–75% B; and 65–70 min, 75–100% B, followed by a 15 min hold time. Chromatographic conditions were maintained at 25 °C with a flow rate of 1 mL/min, an injection volume of 10 µL, and detection at 254 nm.

Qualitative and quantitative analyses were conducted by comparing retention times and UV spectra with those of authentic standards. The reference compounds included 29 phenolic acids: caffeic (y = 59811859.92x − 145632.27, R^2^ = 0.9998), 4-*O*-caffeoylquinic, caftaric, chlorogenic (y = 44790418.33x − 45222.51, R^2^ = 0.9998), *m*-coumaric, *o*-coumaric (y = 96831961.16x − 4785.87, R^2^ = 0.9999), *p*-coumaric, cryptochlorogenic, 1,5-, 3,4-, and 3,5-dicaffeoylquinic, 3,4-dihydroxyphenylacetic, ellagic, ferulic, 4-feruloylquinic, gallic, gentisic, hydrocaffeic (y = 5883705.01x + 30869.71, R^2^ = 0.9999), *p*-hydroxybenzoic, isochlorogenic, isoferulic (y = 8327907.76x − 1247593.21, R^2^ = 0.9945), neochlorogenic, phenylacetic, protocatechuic, rosmarinic, salicylic, sinapic, syringic (y = 62895357.59x + 57381.90, R^2^ = 0.9994), and vanillic acids—as well as their precursors (benzoic and cinnamic acids). Additionally, 24 flavonoid standards were used: apigenin, apigenin-7-glucuronide, astragalin, avicularin, cymaroside, chrysin, guaiaverin, hyperoside, isoquercitrin (y = 88387200.22x + 112999.96, R^2^ = 0.9999), kaempferol, kaempferol-4-glucoside, kaempferol-7-rhamnoside, quercetin (y = 67269930.43x − 813339.41, R^2^ = 0.9990), quercimetrin, quercitrin, luteolin, myricetin (y = 44643051.91x − 1581300.73, R^2^ = 0.9996), naringin, populin, rhamnetin, robinin, rutin, trifolin (y = 80650805.89x-583711.04, R^2^ = 0.9997), vitexin and also 14 isoflavones: biochanin A, calycosin, calycosin 7-*O*-glucoside, daidzein, formonoetin, genistein, genistin, glycitin, hesperetin, hesperidin, ononin, pseudobaptigenin, rothindin, taxifolin. All standards were purchased from Sigma-Aldrich (Saint Louis, MO, USA). Quantification used external calibration curves. According to the validated protocol [51], limits of detection (LOD) ranged from 0.007 to 0.024 mg/mL, and limits of quantification (LOQ) from 0.023 to 0.072 mg/mL.

### 4.8. Determination of Sterol and Triterpenoid Compounds by HPLC-DAD

The quantitative analyses of sterol and triterpenoid compounds were performed using a modified HPLC-DAD method based on previously published procedures [53]. Ethanolic extracts prepared as described above were analyzed using a Merck-Hitachi LaChrom Elite HPLC system (Merck KGaA, Darmstadt, Germany) equipped with a DAD L-2455 detector and a Purospher RP-18e analytical column (250 × 4 mm, 5 μm; Merck, Germany). The mobile phase consisted of methanol (A) and acetonitrile (B). Chromatographic separation was carried out under isocratic conditions using 30% A and 70% B for 60 min. The flow rate was maintained at 1.0 mL/min, the column temperature was set at 25 °C, and the injection volume was 10 μL. Detection was performed at 210 nm.

Qualitative identification of the analyzed compounds was achieved by comparing retention times and UV spectra with those of authentic reference standards. The following reference compounds were used: β-sitosterol (y = 17088874.58x + 129511.75, R^2^ = 0.9999), daucosterol (y = 9245075.96x + 17433.67, R^2^ = 0.9999), α-spinasterol, brassicasterol, stigmasterol, and ursolic acid (y = 18038749.01x + 114169.29, R^2^ = 0.9999) (Sigma-Aldrich, St. Louis, MO, USA). Quantitative determination of β-sitosterol, daucosterol, and ursolic acid was performed using the external calibration curve method. The concentrations of the analyzed compounds were expressed as mg/100 g DW.

### 4.9. Assessment of Biological Activities

#### 4.9.1. DPPH Radical-Scavenging Assay

The DPPH radical-scavenging activity was determined using a modified microplate method based on Brand-Williams et al. [54]. A 0.125 mM DPPH solution was prepared in ethanol. Extracts dissolved in 70% ethanol (2 mg/mL) were serially diluted, and 100 µL of each dilution was mixed with 100 µL of DPPH solution in a 96-well plate. After incubation for 10 min in the dark, absorbance was measured at 540 nm using a SPECTROstar Nano microplate reader (BMG LABTECH GmbH, Ortenberg, Germany). Sample blanks contained the extract solution and ethanol, while the control contained ethanol and DPPH solution. Vitamin C (Sigma-Aldrich, St. Louis, MO, USA) was used as a positive control. Each of the three independent biological replicates was analyzed in three technical replicates.

DPPH scavenging activity was calculated as:

$$\mathrm{DPPH}\;\mathrm{scavenging}\;\mathrm{activity}\;(\%)\;=\;[1\;-\;\frac{{Abs}_{s}\;-\;{Abs}_{b}}{{Abs}_{c}}]\times 100,$$

where *Abs*_s_ is the absorbance of the sample, *Abs_b_* is the absorbance of the corresponding sample blank and *Abs_c_* is the absorbance of the control.

#### 4.9.2. ABTS Radical-Scavenging Assay

The ABTS radical-scavenging activity was determined using a modified microplate method based on Re et al. [55]. A 7 mM ABTS solution and a 2.45 mM potassium persulfate solution were prepared in deionized water. Aliquots of both solutions (2 mL each) were mixed and incubated for 12 h at room temperature in the dark to generate the ABTS^•+^ radical cation. Subsequently, the solution was diluted with 11.5 mL of absolute ethanol and 34.5 mL of deionized water.

The extracts were prepared in 70% ethanol at an initial concentration of 10 mg/mL and serially diluted. An aliquot of each extract dilution (10 µL) was mixed with 190 µL of ABTS^•+^ solution in a 96-well plate. After incubation for 15 min at room temperature in the dark, absorbance was measured at 630 nm using a SPECTROstar Nano microplate reader (BMG LABTECH GmbH, Ortenberg, Germany). The control contained 10 µL of 70% ethanol and 190 µL of ABTS^•+^ solution. Vitamin C was analyzed under the same experimental conditions and used as a positive control. Each of the three independent biological replicates was analyzed in three technical replicates.

ABTS radical-scavenging activity was calculated as follows:

$$\mathrm{ABTS}\;\mathrm{scavenging}\;\mathrm{activity}\;(\%)\;=\;[1\;-\;\frac{{Abs}_{s}\;-\;{Abs}_{b}}{{Abs}_{c}}]\times 100,$$

where *Abs_s_* is the absorbance of the sample, *Abs_b_* is the absorbance of the corresponding sample blank and *Abs_c_* is the absorbance of the control.

#### 4.9.3. Tyrosinase Inhibitory Assay

The mushroom tyrosinase inhibitory activity was determined using a modified microplate method based on Wang et al. [56]. The extracts were initially dissolved in DMSO (WARCHEM Sp. z o.o., Zakręt, Poland) and subsequently diluted with phosphate buffer (100 mM, pH 6.8) to prepare working solutions at concentrations ranging from 0.5 to 2 mg/mL, corresponding to final concentrations of 0.05–0.2 mg/mL in the reaction mixture (total volume: 200 µL). Kojic acid (Sigma-Aldrich, St. Louis, MO, USA), prepared at the same concentrations, was used as a positive control.

Aliquots of the extracts (20 µL) were mixed with 120 µL of phosphate buffer and 20 µL of mushroom tyrosinase solution (500 U/mL; Sigma-Aldrich, St. Louis, MO, USA) in a 96-well plate. After incubation for 10 min at room temperature, 40 µL of 4 mM L-DOPA (Sigma-Aldrich, St. Louis, MO, USA) was added. The mixtures were incubated for a further 20 min at room temperature, and absorbance was measured at 450 nm using a SPECTROstar Nano microplate reader (BMG LABTECH GmbH, Ortenberg, Germany).

Sample blanks contained 20 µL of the extract solution and 180 µL of phosphate buffer. The absorbance of each sample blank was subtracted from the corresponding sample absorbance to correct for background absorbance associated with the plant extracts. The control, representing 100% tyrosinase activity, was prepared by replacing the extract with a corresponding DMSO solution diluted in phosphate buffer. The solvent control contained the same final concentration of DMSO as the extract-treated samples to account for potential solvent effects on enzyme activity. Each of the three independent biological replicates was analyzed in three technical replicates.

Tyrosinase inhibition (%) was calculated as follows:

$$\mathrm{Tyrosinase}\;\mathrm{inhibition}\;(\%)\;=\;[1\;-\;\frac{{Abs}_{s}\;-\;{Abs}_{b}}{{Abs}_{c}}]\times 100,$$

where *Abs_s_* is the absorbance of the sample, *Abs_b_* is the absorbance of the corresponding sample blank and *Abs_c_* is the absorbance of the control representing 100% tyrosinase activity.

#### 4.9.4. Elastase Inhibitory Assay

The elastase inhibitory activity was determined using a modified microplate method based on Thring et al. [57]. The extracts were initially dissolved in DMSO and subsequently diluted with 100 mM Tris-HCl (Thermo Fisher Scientific, Waltham, MA, USA) buffer (pH 8.0) to prepare working solutions at concentrations of 1 and 2 mg/mL, corresponding to final concentrations of 0.1 and 0.2 mg/mL in the reaction mixture (total volume: 150 µL). Epigallocatechin (Sigma-Aldrich, St. Louis, MO, USA), tested under the same experimental conditions, was used as a positive control.

Aliquots (15 µL) of each extract solution were mixed with 90 µL of Tris-HCl buffer and 25 µL of elastase working solution (0.05 mg/mL, corresponding to 0.2 U per well; Sigma-Aldrich, St. Louis, MO, USA) in a 96-well plate. After pre-incubation for 10 min at room temperature, 20 µL of 2.9 mM N-succinyl-Ala-Ala-Ala-p-nitroanilide (SANA; Sigma-Aldrich, St. Louis, MO, USA) was added. The reaction mixtures were incubated for 20 min at 37 °C, and absorbance was measured at 405 nm using a SPECTROstar Nano microplate reader (BMG LABTECH GmbH, Ortenberg, Germany).

Sample blanks contained 15 µL of the corresponding extract solution, 25 µL of elastase solution, and 110 µL of Tris-HCl buffer without SANA. The absorbance of each sample blank was subtracted from the corresponding sample absorbance to correct for background absorbance associated with the plant extracts. The control, representing 100% elastase activity, contained 105 µL of Tris-HCl buffer, 25 µL of elastase solution, and 20 µL of SANA. Additionally, a solvent control was prepared by replacing the extract with a corresponding DMSO solution diluted in Tris-HCl buffer, maintaining the same final DMSO concentration as in the extract-treated samples. Each of the three independent biological replicates was analyzed in three technical replicates.

The percentage of elastase inhibition was calculated using the following equation:

$$\mathrm{Elastase}\;\mathrm{inhibition}\;(\%)\;=\;[1\;-\;\frac{{Abs}_{s}\;-\;{Abs}_{b}}{{Abs}_{c}}]\times 100,$$

where Abs_s_ is the absorbance of the sample, Abs_b_ is the absorbance of the corresponding sample blank and Abs_c_ is the absorbance of the control representing 100% elastase activity.

#### 4.9.5. Anti-Microbial Assay

The antimicrobial activity of the tested dried extract was evaluated against a panel of reference microorganisms, including the fungal strain *Candida albicans* ATCC 10231 and the bacterial strains *Streptococcus mutans* ATCC 25175, *Staphylococcus epidermidis* ATCC 8853, *Streptococcus agalactiae* PCM 2683, *Staphylococcus aureus* 6538P, *Staphylococcus epidermidis* PCM 2118, *Escherichia coli* ATCC 8739, and *Pseudomonas aeruginosa* PAO1.

A modified broth microdilution assay based on EUCAST principles was used [58]. Dried extracts were dissolved in DMSO and diluted in Mueller–Hinton broth to obtain twofold serial dilutions with final concentrations of 0.3125–20 mg/mL. The final DMSO concentration did not exceed 1% (*v*/*v*). The initial microbial density in the assay wells corresponded to an absorbance of 0.1 at 600 nm, excluding the contribution of the extracts. *C. albicans* was initially cultured on Sabouraud medium, whereas extract testing was performed in Mueller–Hinton broth for all microorganisms.

Microplates were incubated at 37 °C for 24 h, and absorbance was measured at 600 nm using an Infinite M200 PRO microplate reader (Tecan, Männedorf, Switzerland). Uninoculated controls containing the corresponding extract concentrations were included to assess extract-derived colour and turbidity. Sample readings were evaluated against these matched extract controls; background absorbance was not numerically subtracted.

To support endpoint interpretation, samples from successive wells were plated onto Mueller–Hinton agar and incubated overnight at 37 °C. Colonies were counted where countable. The minimum inhibitory concentration (MIC) was operationally defined as the lowest tested extract concentration at which no detectable growth above the corresponding extract background was observed, with supplementary plating results used to resolve ambiguous readings. Each of the three independent biological replicates was analyzed in three technical replicates.

### 4.10. Statistical Analysis

Statistical analyses were performed using three independent biological replicates (*n* = 3) for each cultivation period. Data are presented as mean ± standard deviation (SD). Statistical analyses were conducted using Statistica13 software (StatSoft, Kraków, Poland). Differences among experimental groups were evaluated using one-way analysis of variance (ANOVA), followed by Tukey’s HSD post hoc test for multiple comparisons. Differences were considered statistically significant at *p* < 0.05. Technical replicate measurements were averaged within each biological replicate prior to statistical analysis and were not treated as independent observations.

The Principal Component Analysis (PCA) was performed with PAST 4.03 [59]. Cluster analysis was conducted to identify hierarchical relationships, with results visualized as dendrograms combined with heatmaps using the Heatmapper tool [60]. PCA and heatmaps were based on individual biological replicate-level data, with each analysis comprising 15 samples representing five experimental groups with three biological replicates each. Two separate PCAs and two corresponding heatmaps were generated: one set for phytochemical composition and enzyme-inhibition activity, and the other for ABTS–DPPH radical-scavenging responses. The phytochemical and biological activity heatmap included 22 variables, whereas the corresponding PCA included 15 variables with complete numerical data across all 15 samples. The ABTS–DPPH heatmap included 17 variables, comprising eight ABTS and nine DPPH responses, whereas the corresponding PCA included 12 variables with complete numerical data, comprising eight ABTS and four DPPH responses. Before each PCA, variables were independently standardized using z-scores (mean-centering and scaling to unit standard deviation) across all 15 samples to account for differences in measurement units and numerical ranges. PCA was performed on the covariance matrix of the standardized data. The phytochemical and biological activity heatmap displayed column-wise z-score-standardized values, whereas the ABTS–DPPH heatmap displayed radical inhibition percentages. Hierarchical clustering was used to explore similarities among samples and variables. PCA and clustering were considered exploratory analyses.

## 5. Conclusions

This study provides the first comprehensive insight into the phytochemical composition and biological potential of *L. germanicus* extracts obtained from the parent plant and in vitro microshoot cultures. The extracts contained diverse secondary metabolites, including flavonoids, phenolic acids, sterol-related compounds, and pentacyclic triterpenes. Parent-plant extracts generally showed higher metabolite contents and stronger antioxidant and enzyme-inhibitory activities, whereas the phytochemical profile and biological activity of microshoot cultures varied with cultivation time.

The extracts also exhibited selective antibacterial activity, mainly against Gram-positive bacteria, with the lowest MIC value (5 mg/mL) observed against *S. aureus*. Despite their lower overall metabolite levels, the microshoot cultures retained measurable biological activity, supporting their potential as an alternative source of bioactive plant material.

Overall, *L. germanicus* represents a potential source of bioactive compounds for further investigation in cosmetic applications, particularly in relation to oxidative stress, skin aging, pigmentation, and microbial imbalance. However, the present findings are preliminary, as the biological activities were evaluated using in vitro assays without confirmation in skin-relevant models or formulated cosmetic products. Further studies should focus on optimization of in vitro culture conditions and evaluation of cytotoxicity, skin compatibility, and biological efficacy using skin-relevant cellular models and appropriately formulated cosmetic products.

## Figures and Tables

**Table 1:** Total polyphenol content (TPC) (mg GAE/100 g DW ± SD), total flavonoid content (TFC) (mg RE/100 g DW) and concentrations of individual phenolic compounds, sterols, and triterpenoids (mg/100 g DW ± SD) in *L. germanicus* extracts from leaf, herb and microshoots agar cultures. Results are expressed as mean ± standard deviation (SD) of three independent biological replicates (*n* = 3).

Group of Compounds	Compound	In Vitro Cultures			Parent Plant	
		10 Days	20 Days	30 Days	Leaf	Herb
**Phenolic** **acids**	Caffeic acid	32.74 ± 7.05 ^b^	27.61 ± 1.89 ^bc^	51.06 ± 2.73 ^a^	13.54 ± 2.98 ^c^	22.61 ± 9.64 ^bc^
	Chlorogenic acid	nd *	nd	nd	26.08 ± 4.63 ^b^	41.61 ± 5.47 ^a^
	*o*-Coumaric acid	nd	nd	nd	18.49 ± 1.16 ^b^	39.27 ± 4.12 ^a^
	Hydrocaffeic acid	nd	nd	nd	417.01 ± 24.43 ^b^	575.53 ± 44.38 ^a^
	Isoferulic acid	25.80 ± 12.77 ^a^	13.22 ± 5.79 ^a^	22.08 ± 5.02 ^a^	nd	nd
	Syringic acid	0.40 ± 0.38 ^b^	0.29 ± 0.27 ^b^	1.56 ± 1.48 ^b^	36.44 ± 10.07 ^a^	41.91 ± 7.36 ^a^
**Flavonoids**	Isoquercitrin	25.11 ± 16.33 ^c^	4.61 ± 3.21 ^c^	4.92 ± 1.95 ^c^	5057.90 ± 91.24 ^b^	5860.00 ± 106.03 ^a^
	Myricetin	7.60 ± 7.60 ^bc^	1.82 ± 0.72 ^c^	3.47 ± 1.57 ^bc^	26.09 ± 12.49 ^b^	280.14 ± 11.99 ^a^
	Trifolin	nd	nd	nd	716.46 ± 30.52 ^b^	928.50 ± 18.49 ^a^
	Quercetin	3.46 ± 4.18 ^c^	1.82 ± 1.72 ^c^	1.27 ± 0.58 ^c^	70.39 ± 4.75 ^b^	148.05 ± 6.70 ^a^
**Sterol and triterpenoid compounds**	Daucosterol	37.10 ± 4.00 ^c^	59.97 ± 3.43 ^bc^	82.61 ± 9.53 ^b^	230.81 ± 4.80 ^a^	202.78 ± 22.53 ^a^
	β-Sitosterol	70.03 ± 3.68 ^bc^	92.47 ± 5.06 ^b^	152.94 ± 8.98 ^a^	54.42 ± 22.71 ^cd^	39.52 ± 2.47 ^d^
	Ursolic acid	88.06 ± 9.94 ^b^	53.78 ± 5.59 ^c^	146.30 ± 13.76 ^a^	75.29 ± 0.84 ^bc^	58.46 ± 5.22 ^c^
**Total polyphenol content (TPC)**		27.85 ± 1.90 ^b^	32.32 ± 2.83 ^b^	32.98 ± 4.38 ^b^	228.52 ± 44.11 ^a^	260.98 ± 32.67 ^a^
**Total flavonoid content (TFC)**		37.22 ± 4.37 ^d^	60.71 ± 4.11 ^c^	47.22 ± 9.34 ^cd^	1033.75 ± 59.22 ^a^	723.61 ± 36.29 ^b^

* nd—not detected; different letters indicate significant differences between the tested extracts (*p* < 0.05);

**Table 2:** Anti-microbial activity of *L. germanicus* extracts from leaf, herb and microshoot cultures.

Microorganism		Leaf	Herb	Microshoot Cultures		
				10 Days	20 Days	30 Days
		MIC (mg/mL)				
**Gram-** **positive** **bacteria**	*Staphylococcus aureus* 6538P	>20	>20	>20	10	5
	*Staphylococcus* *epidermidis* ATCC 8853	>20	>20	>20	20	>20
	*Staphylococcus* *epidermidis* PCM 2118	>20	>20	>20	20	>20
	*Streptococcus agalactiae* PCM 2683	>20	>20	>20	20	>20
	*Streptococcus mutans* ATCC 25175	>20	20	20	20	20
**Gram-** **negative** **bacteria**	*Escherichia coli* ATCC 8739	>20	>20	>20	>20	>20
	*Pseudomonas* *aeruginosa* PAO1	>20	>20	>20	>20	>20
**Fungi**	*Candida albicans*	>20	>20	>20	>20	>20

## Data Availability

Data are contained within the article.

## Abbreviations

The following abbreviations are used in this manuscript:

HPLC-DAD	High-Performance Liquid Chromatography with Diode-Array Detection
BA	6-benzyladenine
NAA	1-naphthaleneacetic acid
GA_3_	gibberellic acid
TPC	total polyphenol content
GAE	gallic acid equivalent
DW	dried weight
TFC	total flavonoid content
RE	rutin equivalent
ABTS	2,2′-azinobis(3-ethylbenzothiazoline-6-sulfonic acid
DPPH	2,2-diphenyl-1-picrylhydrazyl
IC_50_	half-maximal inhibitory concentration
MIC	minimum inhibitory concentration

## References

[1] Slavik B. (1995). A Plant-geographical study of the genus *Dorycnium* Mill. (Fabaceae) in the Czech Republic. Folia Geobot. Phytotax.

[2] Joshi P., Kramina T.E., Lysova M.V., Samigullin T.H., Özbek M.U., Sokoloff D.D. (2022). When morphology and biogeography approximate nuclear ITS but conflict with plastid phylogeny: Phylogeography of the *Lotus dorycnium* Species Complex (Leguminosae). Plants.

[3] Herre I., Zidorn C. (2025). Phytochemistry of the Tribe Loteae (Fabaceae, Faboideae). Biochem. Syst. Ecol..

[4] Infoflora. https://www.infoflora.ch/en/flora/dorycnium-germanicum.html.

[5] Rote Liste Zentrum. https://www.rote-liste-zentrum.de/en/detailseite/?species_uuid=8c4de8be-848c-41bd-8ed6-594313f43a09.

[6] Pladias. https://pladias.cz/en/taxon/data/Dorycnium%20germanicum.

[7] Kaźmierczakowa R., Bloch-Orłowska J., Celka Z., Cwener A., Dajdok Z., Michalska-Hejduk D., Pawlikowski P., Szczęśniak E., Ziarnek K. (2016). Polish Red List of Pteridophytes and Flowering Plants.

[8] Pistelli L., Noccioli C., Martera M., Giamperi L., Bucchini A., Fraternale D., Ricci D. (2006). Antioxidant flavonoids glycosides from *Dorycnium hirsutum*. Chem. Nat. Compd..

[9] Hanganu D., Vlase L., Olah N. (2010). Phytochemical Analysis of isoflavons from some Fabaceae species extracts. Not. Bot. Horti Agrobot. Cluj Napoca.

[10] Moreno A., Martin-Cordero C., Iglesias-Guerra F., Toro M.V. (2002). Flavonoids from *Dorycnium rectum*. Biochem. Syst. Ecol..

[11] Sivakumaran S., Molan A.L., Meagher L.P., Kolb B., Foo L.Y., Lane G.A., Attwood G.A., Fraser K., Tavendale M. (2004). Variation in antimicrobial action of proanthocyanidins from *Dorycnium rectum* against rumen bacteria. Phytochemistry.

[12] Movsumov I.S., Yusifova D.Y., Suleymanov T.A., Garaev E.A. (2020). Biologically active compounds from *Dorycnium graecum* growing in Azerbaijan. Chem. Nat. Compd..

[13] Molan A.L., Waghorn G.C., Min B.R., Mcnabb W.C. (2000). The effect of condensed tannins from seven herbages on *Trichostrongylus colubriformis* larval migration in vitro. Folia Parasitol..

[14] Usta C., Yildirim A.B., Turker A.U. (2014). Antibacterial and antitumour activities of some plants grown in Turkey. Biotechnol. Biotechnol. Equip..

[15] Tosun F., Albayrak S., Acar Şah E., Çeçen Ö. (2024). Phytochemical profile and antioxidant activity potential of *Lotus sanguineus* (Vural) D.D.Sokoloff. Acta Pharm. Sci..

[16] Stefanović O.D., Tešić J.D., Čomić L.R. (2015). *Melilotus albus* and *Dorycnium herbaceum* extracts as source of phenolic compounds and their antimicrobial, antibiofilm, and antioxidant potentials. J. Food Drug Anal..

[17] Koygun G., Arslan E., Zengin G., Orlando G., Ferrante C. (2021). Comparison of anticancer activity of *Dorycnium pentaphyllum* extract on Mcf-7 and Mcf-12a cell line: Correlation with invasion and adhesion. Biomolecules.

[18] Waghorn T., Molan A., Deighton M., Alexander R., Leathwick D., McNabb W., Meagher L. (2006). In vivo anthelmintic activity of *Dorycnium rectum* and grape seed extract against *Ostertagia* (*Teladorsagia*) *circumcincta* and *Trichostrongylus colubriformis* in Sheep. N. Z. Vet. J..

[19] Nicolaescu O., Varut R.-M., Patruna S. (2019). Determination of the polyphenolic content of *Dorycnium herbaceum* plant extract and testing in vivo antioxidant effect, comparative with bilberry. Rev. De Chim..

[20] Pieroni A., Giusti M.E., de Pasquale C., Lenzarini C., Censorii E., Gonzáles-Tejero M., Sánchez-Rojas C., Ramiro-Gutiérrez J.M., Skoula M., Johnson C. (2006). Circum-mediterranean cultural heritage and medicinal plant uses in traditional animal healthcare: A field survey in eight selected areas within the RUBIA project. J. Ethnobiol. Ethnomed..

[21] González-Tejero M.R., Casares-Porcel M., Sánchez-Rojas C.P., Ramiro-Gutiérrez J.M., Molero-Mesa J., Pieroni A., Giusti M.E., Censorii E., de Pasquale C., Della A. (2008). Medicinal plants in the Mediterranean area: Synthesis of the results of the project Rubia. J. Ethnopharmacol..

[22] Rigat M., Bonet M.À., Garcia S., Garnatje T., Vallès J. (2007). Studies on pharmaceutical ethnobotany in the High River Ter Valley (Pyrenees, Catalonia, Iberian Peninsula). J. Ethnopharmacol..

[23] Bremner P., Rivera D., Calzado M.A., Obón C., Inocencio C., Beckwith C., Fiebich B.L., Muñoz E., Heinrich M. (2009). Assessing medicinal plants from South-Eastern Spain for potential anti-inflammatory effects targeting nuclear factor-kappa B and other pro-inflammatory mediators. J. Ethnopharmacol..

[24] Szopa A., Dziurka M., Granica S., Klimek-Szczykutowicz M., Kubica P., Warzecha A., Jafernik K., Ekiert H. (2020). *Schisandra rubriflora* plant material and in vitro microshoot cultures as rich sources of natural phenolic antioxidants. Antioxidants.

[25] Rajesh M., Sivanandhan G., Jeyaraj M., Chackravarthy R., Manickavasagam M., Selvaraj N., Ganapathi A. (2014). An efficient in vitro system for somatic embryogenesis and podophyllotoxin production in *Podophyllum hexandrum* Royle. Protoplasma.

[26] Turker H. (2025). Investigation of the physiological and antioxidant properties of the medicinal and endemic *Hypericum bilgehan-bilgilii* species under different cultivation methods. Biology.

[27] Shraim A.M., Ahmed T.A., Rahman M.M., Hijji Y.M. (2021). Determination of total flavonoid content by aluminum chloride assay: A critical evaluation. LWT.

[28] Enchev P., Zarev Y., Marinov L., Rivera-Mondragón A., Ionkova I. (2024). Antiproliferative activity of extracts containing flavonoids from in vitro callus cultures of *Astragalus onobrychis*. Rev. Bras. De Farmacogn..

[29] Gonçalves J.C., Coelho M.T., Da Graça Diogo M., Alves V.D., Rosário Bronze M., Coimbra M.A., Martins M., Moldão-Martins M. (2018). In vitro shoot cultures of *Pterospartum tridentatum* as an alternative to wild plants as a source of bioactive compounds. Nat. Prod. Commun..

[30] Kokotkiewicz A., Wnuk M., Bucinski A., Luczkiewicz M. (2009). In vitro cultures of *Cyclopia* Plants (Honeybush) as a source of bioactive xanthones and flavanones. Z. Fur Naturforschung-Sect. C J. Biosci..

[31] Klimek-Szczykutowicz M., Nowak A., Muzykiewicz-Szymańska A., Kucharski Ł., Lechwar P., Gaweł-Bęben K., Wiśniewska K., Piwowarczyk R., Kokotkiewicz A., Luczkiewicz M. (2026). Comparative phytochemical and biological profiling of *Trifolium rubens* L. leaves, flowers, and callus cultures for cosmetic applications. Sci. Rep..

[32] Wołosiak R., Drużyńska B., Derewiaka D., Piecyk M., Majewska E., Ciecierska M., Worobiej E., Pakosz P. (2022). Verification of the conditions for determination of antioxidant activity by ABTS and DPPH assays—A practical approach. Molecules.

[33] Bashir A., Munir A., Hira H., Akram R., Ghafoor A., Mustafa A.B., Jamee M. (2025). Green guardians: Plant-derived antioxidants and their Role in oxidative stress control. Agrobiol. Rec..

[34] Alharthi N.S. (2025). Prophylactic Impacts of lotusine against hyperglycaemia-induced oxidative stress in hepatic cells isolated from diabetic rats via Irs-1/Pi3 K/Akt pathway. Pak. Vet. J..

[35] Placines C., Castaneda-Loaiza V., Rodrigues M.J., Pereira C.G., da Silva J.P., Zengin G., Custódio L. (2021). In vitro enzyme inhibitory and antioxidant properties, cytotoxicity, and LC-DAD-ESI-MS/MS profile of extracts from the halophyte *Lotus creticus* L. Jundishapur J. Nat. Pharm. Prod..

[36] Lou Y., Xu T., Cao H., Zhao Q., Zhang P., Shu P. (2022). Natural antioxidants, tyrosinase and acetylcholinesterase inhibitors from *Cercis glabra* leaves. Molecules.

[37] Maisto M., Piccolo V., Marzocchi A., Ricci L., Romano B., Maresca D.C., Di Lorenzo R., Laneri S., Ercolano G., Ianaro A. (2026). Apple oil as a source of ursolic acid for the treatment of hyperpigmentary disorders with molecular and clinical evaluation. Sci. Rep..

[38] Theansungnoen T., Nitthikan N., Wilai M., Chaiwut P., Kiattisin K., Intharuksa A. (2022). Phytochemical analysis and antioxidant, antimicrobial, and antiaging activities of ethanolic seed extracts of four *Mucuna* species. Cosmetics.

[39] Huang Y., Chen L., Feng L., Guo F., Li Y. (2013). Characterization of total phenolic constituents from the stems of *Spatholobus suberectus* using LC-DAD-MS and their inhibitory effect on human neutrophil elastase activity. Molecules.

[40] Schmitt M., Magid A.A., Etique N., Hubert J., Duca L., Nuzillard J.-M., Voutquenne-Nazabadioko L. (2022). Bio-guided studies of *Lotus maritimus* aerial parts and investigation of their antioxidant, tyrosinase and elastase inhibitory activities. Nat. Prod. J..

[41] Feng L., Liu X., Zhu W., Guo F., Wu Y., Wang R., Chen K., Huang C., Li Y. (2013). Inhibition of human neutrophil elastase by pentacyclic triterpenes. PLoS ONE.

[42] Rodjan P., Kaewnabon Y., Chimplee S., Pongpom M., Tedja I., Mitsuwan W., Jeenkeawpieam J. (2025). Antifungal potential and safety evaluation of thai *Piper betle* leaf extract and phenolics against animal pathogenic *Candida* species. Pak. Vet. J..

[43] Ullah U., Mumtaz M.Z., Jaffar T.H., Khaskheli M.A., Imran A., Zaib A.A., Shehzad S., Pervaiz H., Bibi A., Malik B. (2025). Phytochemical constituents with antimicrobial activity from ethanol extract of *Lawsonia intermis* leaves and stem extracts. Agrobiol. Rec..

[44] Altarjami L.R. (2025). Anticancer and antioxidant activities of polyphenolic pomegranate peel extracts obtained by a novel hybrid ultrasound-microwave method: In vitro and in vivo studies in albino mice with HeLa, Colon, and HepG2 cancerous cell lines. Pak. Vet. J..

[45] Rozporządzenie Ministra Środowiska z dnia 9 października 2014 r. w sprawie ochrony gatunkowej roślin [Regulation of the Minister of the Environment of 9 October 2014 on the Protection of Plant Species]. Journal of Laws of the Republic of Poland 2014, Item 1409. https://eli.gov.pl/eli/DU/2014/1409/ogl.

[46] Kaźmierczakowa R., Zarzycki K., Mirek Z. (2014). Polska Czerwona Księga Roślin. Paprotniki I Rośliny Kwiatowe [Polish Red Data Book of Plants. Pteridophytes and Flowering Plants].

[47] Murashige T., Skoog F. (1962). A revised medium for rapid growth and bioassays with tobacco tissue cultures. Physiol. Plant..

[48] Singleton V., Rossi J. (1965). Colorimetry of total phenolics with phosphomolybdic-phosphotungstic acid reagents. Am. J. Enol. Vitic..

[49] Christ B., Müller K.H. (1960). On the serial determination of the content of flavonol derivatives in drugs. Arch. Pharm..

[50] Sari K.R.P., Ikawati Z., Danarti R., Hertiani T. (2023). Micro-titer plate assay for measurement of total phenolic and total flavonoid contents in medicinal plant extracts. Arab. J. Chem..

[51] Sułkowska-Ziaja K., Maślanka A., Szewczyk A., Muszyńska B. (2017). Physiologically active compounds in four species of genus *Phellinus*. Nat. Prod. Commun..

[52] Ellnain-Wojtaszek M., Zgórka G. (1999). High-Performance Liquid Chromatography and Thin-Layer Chromatography of phenolic acids from *Gingko biloba* L. leaves collected within vegetative period. J. Liq. Chromatogr. Relat. Technol..

[53] Kim J., Kim J.H., Bang S.I., Shin H., Cho E.J., Lee S. (2022). Antioxidant activity of edible sprouts and phytosterol contents by HPLC/UV analysis. Hortic. Environ. Biotechnol..

[54] Brand-Williams W., Cuvelier M., Berset C. (1995). Use of a free radical method to evaluate antioxidant activity. LWT-Food Sci. Technol..

[55] Re R., Pellegrini N., Proteggente A., Pannala A., Yang M., Rice-Evans C. (1999). Antioxidant activity applying an improved ABTS radical cation decolorization assay. Free Radic. Biol. Med..

[56] Wang W., Gao Y., Wang W., Zhang J., Yin J., Le T., Xue J., Engelhardt U.H., Jiang H. (2022). Kojic acid showed consistent inhibitory activity on tyrosinase from mushroom and in cultured B16F10 cells compared with arbutins. Antioxidants.

[57] Thring T.S.A., Hili P., Naughton D.P. (2009). Anti-collagenase, anti-elastase and anti-oxidant activities of extracts from 21 plants. BMC Complement. Altern. Med..

[58] EUCAST. http://www.eucast.org.

[59] Hammer Ø., Harper D.A.T., Ryan P.D. (2001). PAST: Paleontological Statistics Software Package for Education and Data Analysis. Palaeontol. Electron..

[60] Babicki S., Arndt D., Marcu A., Liang Y., Grant J.R., Maciejewski A., Wishart D.S. (2016). Heatmapper: Web-enabled heat papping for all. Nucleic Acids Res..

